# Primary Productivity and Habitat Depth Shape Developmental Mode in European Marine Gastropods

**DOI:** 10.1002/ece3.73147

**Published:** 2026-03-08

**Authors:** Nicolás Weidberg, Juan Bueno‐Pardo, Ainhoa de Diego, José Luis Acuña

**Affiliations:** ^1^ Department of Organisms and Systems Biology University of Oviedo Oviedo Spain; ^2^ Marine Research Centre (CIM), Future Oceans Lab University of Vigo Vigo Spain

**Keywords:** dispersal, larval development, marine biogeography, metabolic theory of ecology, primary productivity, Thorson's rule

## Abstract

Prolonged larval development in marine gastropods is less frequent in high latitudes, with non‐pelagic larval development much more common in these regions. This pattern has been historically referred to in biogeography as the Thorson's rule. The most invoked theoretical explanation for this pattern is that pelagic larval duration becomes too long as temperatures decrease, thus increasing exposure to predators and transport towards habitats not suitable for recruitment. However, more factors rather than only water temperature could influence pelagic duration and overall larval performance, like initial embryo and juvenile sizes, currents and phytoplankton fluctuations. On the other hand, phylogeny and environmental conditions affecting the adults, like the stability of benthic habitats in time, could also play an important role in the evolution of developmental modes. Besides, Thorson's latitudinal pattern could be an artefact arising from insufficient, incomplete and uneven sampling. In this work, we gathered an up‐to‐date dataset for 94 species of European gastropods from the literature, including developmental mode and other life history traits together with variables related to bottom habitat, water column and primary productivity. Thorson's rule was not recovered when the proportion of species with non‐pelagic development was analysed with fine spatial resolution at regions with at least 20 gastropod species present. Moreover, temperature and phylogeny played a negligible role in determining developmental mode, while greater depths and marked chlorophyll‐a seasonality significantly favoured non‐pelagic development. Thus, we infer that the increased temporal shifts in bottom habitats at shallow waters in regions with a non‐seasonal and more constant phytoplankton availability drive the evolution of pelagic larval forms in gastropods.

## Graphical abstract

Thorson's rule suggests that marine gastropods in colder, high‐latitude regions tend to have non‐pelagic (non‐drifting) larval development. However, this study on 94 European gastropod species found that the rule disappears when examined at finer spatial scales, with temperature and phylogeny having little influence. Instead, factors such as greater depth and strong seasonal variation in chlorophyll‐a (a proxy for productivity) were linked to non‐pelagic development, indicating that habitat stability and food availability—not latitude—primarily shape larval strategies.

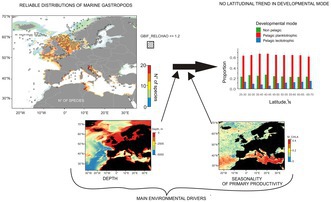

## 1 Introduction

Species distributions in the marine realm and the environmental forcing behind them have become a hot topic in recent years (Barange et al. 2010; Torossian et al. 2016; Davies et al. 2023). Pervasive and accelerated climate change has put into question the ability of marine organisms to cope with the rapid rates of change, especially concerning key eco‐physiological variables like temperature and primary productivity (Brierley and Kingsford 2009; Lima and Wethey 2012; González Taboada and Anadón 2014). Among the possible responses to these challenging new conditions, bathymetric and latitudinal shifts in spatial distributions are frequently regarded as plausible for nektonic, benthonic and planktonic components of the ecosystem, as they have already been recorded worldwide (Kortsch et al. 2015; Chapman et al. 2020; Djeghri et al. 2023). A cue to predict such distribution rearrangements can be provided by biogeographical rules (Tian and Benton 2020). The Island and the Bergmann's rule, among others, summarise the distribution of many different species across a wide range of lineages using a single common trait which they link with a key environmental determinant that varies across a spatial gradient (Welch 2010; Campbell et al. 2021). Because the environmental rates of change can be predicted under different global change scenarios with some confidence, at least on regional scales (Giorgi and Francisco 2000; O'Neill et al. 2020; IPCC 2023), one could simulate the expansion or contraction of a given species distribution by tracking latitudinal shifts of isolines for that environmental variable (Weiss et al. 2018; Lenoir et al. 2020). However, the extent, magnitude, timescale and periodicity of these displacements are very difficult to forecast for any particular species, even when its environmental niche is well understood (Reeve et al. 2014; Seabra et al. 2015; Davies et al. 2023). Furthermore, the spatial patterns from which a given rule was derived may be the result of processes that occurred on evolutionary time scales that are not compatible with the shorter time spans at which anthropogenic global change is affecting biological communities, although there are attempts to reconcile both scales with hybrid species distribution models (Diamond 2018). Finally, because a given spatial pattern can be based on incomplete and/or inconsistent sampling in space and time, the reliability of a biogeographical rule has to be continuously re‐assessed as new methodologies and observations arise, especially if it is to be used to predict future distribution shifts (Tian and Benton 2020).

One of the most invoked biogeographical patterns in marine environments is the so‐called Thorson's rule. In an extensive report, Gunnar Thorson presented a bar graph where the relative proportion of prosobranch gastropod species with pelagic development decreased with respect to those with non‐pelagic development with latitude, by looking at 7 different locations in the North Atlantic (Figure 5 in Thorson 1950). He supported such latitudinal trend with other available observations included in his report and in previous works but not in the bar graph (Thorson 1936, 1940, 1946; Lebour 1945). This pattern was broadly accepted by researchers in the field of marine biogeography, who gave it the status of a rule (Fretter and Graham 1962; Mileikovsky 1971, 1975). Certainly, explanations for the rule were compelling and aligned with the environmental characteristics of the Nordic seas described by marine scientists in the 19th and 20th centuries (Helland‐Hansen and Nansen 1909; Hurdle 1986). Essentially, Thorson explained the prevalence of non‐pelagic gastropod larvae in arctic and subarctic environments because of their poor food conditions and cold temperatures which would hinder pelagic development (Thorson 1950). Further observations along large‐scale latitudinal transects led to an accumulation of exceptions to the rule, especially in the southern hemisphere. In fact, those new surveys and reviews suggested that lecithotrophic rather than non–pelagic larvae prevailed in cold Patagonian and Antarctic waters (Pearse et al. 1991; Pearse 1994; Dayton et al. 2016). Furthermore, non‐pelagic developers were dominant even at low latitudes at the Atlantic coast of South America in marked contrast with the pattern along the Pacific coast, consistent with Thorson's rule (Gallardo and Penchaszadeh 2001). Such contrast between ocean basins has been attributed to a difference in the type of bottom habitats, although the causal link between bottom type and developmental mode was not clarified (Gallardo and Penchaszadeh 2001). Later on, non‐pelagic development was associated with soft bottoms regardless of latitude possibly because of the variations in disturbance rates (Pappalardo et al. 2014). On the other hand, as Thorson (1950) highlighted, those observations did not take into account depth as a basic descriptor of adult habitat, although the same environmental conditions responsible for the prevalence of non‐pelagic developers in Nordic seas (lack of food, cold temperatures) most certainly apply to deep marine regions. Thus, despite contrasting trends in developmental mode with increasing depths found across and off continental shelves (Rex and Waren 1982; Jablonski and Lutz 1983), no attempts were made to disentangle the relative effects of depth and latitude in the global analyses that would follow (Marshall et al. 2012; Pringle et al. 2014).

As the number of observations increased with the years for different regions and taxa, studies focussed on global analyses and started to include the environmental drivers invoked by Thorson in the first place: chlorophyll and temperature. Marshall et al. (2012) observed an increase in planktotrophic species as mean concentrations of chlorophyll‐a increase, suggesting that the apparent lower primary productivity polewards cannot sustain planktotrophic larvae. However, Thorson's original statement was more related to the seasonality of the phytoplankton bloom than to its grand mean, as the main constraint to larval life would be the ‘very limited periods of continuous phytoplankton production’ (Thorson 1936, 1950). On the other hand, Thorson inferred that cold temperatures may result in long larval periods that could negatively affect larval survival (Thorson 1950). Consistently, Marshall et al. (2012) also found a significant decrease of the pelagic mode at lower temperatures worldwide. By the time of these global analyses, the metabolically driven effects of temperature on larval development had already been studied. Specifically, an empirical formulation describing the decrease in developmental times with increasing temperature valid across a wide range of species was tested successfully (O'Connor et al. 2007). In turn, prolonged developmental times in the plankton caused by cold temperatures were linked with high mortality rates caused by predation and larval losses due to offshore transport to unsuitable habitats where settlement cannot take place (Vance 1973; Morgan 1995; Pechenik 1999). Implicitly, this mechanism had to involve another important environmental driver: ocean circulation. Intuitively, for the same larval duration, weaker currents may remove fewer larvae from their settlement sites than strong currents. Such effect was included in different mathematical models describing larval transport and connectivity along coastal stretches (Largier 2003; White et al. 2009). Eventually, it was proposed that, for a local population to persist in time, the number of recruits has to overpass a minimum retention criterion, that is, the ratio between the advective and diffusive transport distances which are in turn a function of current mean velocity and its standard deviation, respectively, averaged over the pelagic life (Byers and Pringle 2006). For the first time, it was possible to assess where the combination of strong water flows and cold temperatures would make connectivity of coastal populations non‐viable along a given poleward‐oriented coastline, thus favouring non‐pelagic developers. Therefore, a mechanistic framework was provided for Thorsón's rule and apparently confirmed by the next global analyses that follow (Marshall et al. 2012; Pringle et al. 2014). The effect of circulation was evaluated this time and a significant decrease in planktonic developers was found as mean current velocity increased (Pringle et al. 2014).

Despite the apparent match between Thorson's rule and the thermally driven constraints captured by the minimum retention criteria, there are alternative evolutionary pathways that could keep the viability of larvae in cold waters. In particular, an increase in fecundity would in turn increase the recruitment rates well above the retention criteria (Byers and Pringle 2006). Moreover, because of the trade‐offs in between the number of offspring and their size, larvae would become more numerous and smaller in this scenario (Gillooly et al. 2002; Bueno and López‐Urrutia 2012; Bueno 2014). Such smaller young larvae would live much less in the water column if the final larval size also decreases, what would be translated in shorter advective distances and in turn in lower values for the retention criteria (Bueno and López‐Urrutia 2012; Bueno 2014). Because growth rates are higher within smaller size ranges, planktonic duration would shorten drastically with a reduction in larval size (Vance 1973; West 2001; Bueno 2014). These concatenated changes can be more or less limited by morphological characteristics in both larvae and adults that could prevent or accelerate the intercalation of pelagic larvae in the life cycles of marine invertebrates (Anderson 1994; Pechenik 1999). Such effects vary depending on the taxonomic clade under study, thus making phylogenetic corrections unavoidable when inspecting shifts in developmental mode and other life history traits (Pappalardo et al. 2014; Pappalardo and Fernández 2013; Ewers‐Saucedo and Pappalardo 2019). Despite the need for considering the effect of shared evolutionary histories in this kind of studies, some research works did not include such corrections in their statistical analyses (Pringle et al. 2014).

Besides morphological adaptations, a wide panoply of larvae display behaviours on different spatiotemporal scales to reduce advective distances and enhance settlement. In essence they swim vertically, thus remaining at water layers that move shorewards or, at least, not offshore (Pineda 1999; Fuchs 2005; Genin et al. 2005; Pringle 2007; Shanks and Shearman 2009, Miller and Morgan 2013; Weidberg et al. 2019). Such depth regulation can only be effective in reducing advection if ocean circulation is strongly stratified, that is, if there are different layers across the water columns flowing in opposing directions with different velocities (Pineda 1999; Weidberg et al. 2015; Hagerty et al. 2018). This dynamic stratification becomes crucial in order to infer the real potential of larvae to escape undesired offshore transport, but it is not captured in the simple retention criteria based on mean velocities and standard deviations and has not been included in any biogeographical study for marine invertebrates.

In summary, the apparent restrictions to larval life arising in cold seas with strong currents can be bypassed on evolutionary time scales by shifts in life history traits and behaviours enhancing larval retention and recruitment. Regardless of the support provided to Thorson's rule by metabolically driven temperature effects, the numerous exceptions found, the limited spatial coverage of the surveys on which it was built, and the exclusion of critical potential environmental determinants of developmental mode (like depth, seasonality of primary productivity and dynamic stratification), never taken into account together, call for a re‐evaluation of this biogeographical paradigm. In this work we took advantage of new bottom habitat and 3D hydrographical models with unprecedented high spatial resolutions coupled with a detailed bibliographic search to revisit the rule for the same taxonomic group at the same ecoregion where it was first enunciated. In particular, we followed four different procedures to evaluate the rule: (1) we re‐built Thorson's latitudinal bar‐graphs based on our new spatially explicit potential species distributions using different methodologies to maximise the reliability of the distribution pattern; (2) we looked for the environmental drivers behind that developmental mode spatial pattern; (3) we addressed the phylogenetic and environmental effects on the multivariate interspecific assemblage which incorporated developmental mode and its associated life history and morphological traits; and (4) we analysed the taxa specific probability of developmental mode occurrence in light of phylogeny and environmental variables. Our results allow us to infer the validity of the rule and delimit the ultimate hydrographical determinants behind the emergence and loss of larval forms in marine gastropods.

## 2 Materials and Methods

### 2.1 Environmental Dataset

A bathymetric raster layer was downloaded from the General Bathymetric Chart of the Oceans (GEBCO, https://www.gebco.net/data_and_products/gridded_bathymetry_data/) for the European seas (25°‐70° N; 25° W‐37° E) with a spatial resolution of 15 arc‐seconds (450 m). Temperature and currents were extracted for the same area from the NRL HYCOM+NCODA GLBu0.08/expt 91.2 (https://www.hycom.org/data/glbu0pt08/expt‐91pt2) with a spatial resolution of 1/12 degree (10 km aprox.), an uneven depth resolution with 40 different levels (every 2 m from 0 to 12 m; every 5 m from 15 to 50 m; every 10 m from 50 to 100 m; every 50 m from 150 to 400 m; every 100 m from 400 to 1000 m; every 500 m from 1500 to 3000 m; and every 1000 m from 3000 to 5000 m, plus 125 and 1250 m) and a time resolution of 3 h from 18 April 2016 to 20 November 2018. This model is based on a complex data assimilation system that incorporates altimetry and sea surface temperatures from satellites together with in situ vertical temperature profiles from XBTs, ARGO floats and moored buoys (Cummings 2005; Cummings and Smedstad 2013). Mean temperatures (T) were obtained as pixel averages for the whole period across all depths. The same procedure was followed to calculate mean currents, but first total currents (U) were obtained as the vector sum of the meridional and zonal components provided by the model. Finally, the dynamic stratification of the water column (DS) was computed as the vector sum of the standard deviations of both meridional and zonal currents. This metric was preferred over just the standard deviation of U as the latter does not capture the vertical variability of the currents because changes in direction are not considered in U. These calculations were carried out in R with libraries vctrs, dplyr and stringr (Wickham et al. 2022, 2023) to obtain data grids for T, U and DS that were interpolated in QGIS 3.4.15 to get the corresponding raster layers with a resolution of 0.1° (11 km aprox).

### 2.2 Primary Productivity Variables

Monthly composites of surface chlorophyll‐a were retrieved as a raster layer from the NPP Aqua Modis L3SMI satellite dataset with a pixel resolution of 4 km from 16‐05‐2003 to 16‐05‐2022 (https://coastwatch.pfeg.noaa.gov/erddap/griddap/erdMH1chlamday.html). Mean (CHLA_mean) and maximal (CHLA_max) chlorophyll‐a concentration were computed for each pixel. To characterise the seasonality in phytoplankton biomass, we calculated the predictability, constancy and contingency for the time series of chlorophyll‐a concentration extracted from each raster pixel (Colwell 1974). Predictability (P_CHLA) is the repeatability of values of the same magnitude in the same period of the year (e.g., months) across multiple years. Constancy (C_CHLA) is the repeatability of values in the same magnitude class along the whole year. It is maximum if all values fall in the same magnitude class so the variable is constant. Contingency (M_CHLA) is the amount of predictable change that the variable experiences during the year. It is maximum when all values for each time period fall within a unique magnitude class specific for each time period so the variable becomes seasonal. Overall predictability *P* is the sum of *C* and M. All three indexes vary between 0 and 1 and were calculated with the function Colwells from the R package hydrostats (Bond 2022) with 11 default equal magnitude classes. Rasters for CHLA_mean, CHLA_max, P_CHLA, M_CHLA y C_CHLA were created in R with packages raster and lubridate (Grolemund and Wickham 2011; Hijmans 2022) and then visualised in QGIS 3.4.15.

### 2.3 Bottom Habitats

The EUSeaMap 2023 Broad‐Scale Predictive Map for Europe was downloaded (ICES Metadata Catalogue) as a shape file with 28 different categories that arise from combinations between bottom vertical zones (infralittoral, circalittoral, bathyal and abyssal) and substrata morphology (sand, mud, rock, coarse sediment and biogenic reef). This product was created by the EMODnet Seabed habitats from its Geology Seabed Substrate data product in conjunction with multiple data sources (Vasquez et al. 2023). The shapefile was re‐projected in a raster layer with a resolution of 300 m approx. in QGIS 3.4.15.

### 2.4 Gastropod Dataset

A bibliographic search was carried out for morphological, habitat and life history traits of European gastropod species. Mode of development was categorised as non‐pelagic if the whole embryonic period occurred within the egg mass/capsules in the benthos; pelagic‐planktotrophic if larvae develops in the plankton actively feeding, mainly on phytoplankton; and pelagic‐lecithotrophic if the planktonic larvae does not feed but lives on the nutritious reserves initially allocated in the egg. Most of the studies found in this search derived developmental mode by performing rearing experiments from plankton trawls and egg collections, but protoconch morphology was also employed. Adult diet was also considered and categorised as carnivorous, omnivorous and herbivorous. Sizes of initial larvae/embryo (IS), first post metamorphic stages or juveniles (JS) and adults (AS) were also considered and used to calculate three different growth ratios: between juvenile and initial sizes (Gr1); between adult and juvenile sizes (Gr2); and between adult and initial sizes (Gr 3). Sizes were assimilated to volumes that were calculated as spherical for IS and JS and cylindrical (Figure S1 in Appendix S1), conical (Figure S2 in Appendix S1), parallelepipedal (Figure S3 in Appendix S1), or semi‐spheroidal (Figure S4 in Appendix S1) for AS, depending on the specific shape of the adult shell for each species. When size ranges were provided from the available literature, then the average was considered a good proxy of mean size. On the other hand, the spatial distribution, depth range and habitat bottom type for each species were also gathered from the literature.

Distribution maps for each species were built in QGIS 3.4.15 from the latitudinal ranges and geographical boundaries extracted from the literature. Within those distribution areas, a grid with a resolution of 0.1° was used to extract all the environmental and primary productivity variables. Then, averages for all these variables were computed within the given taxa‐specific depth range, thus obtaining mean environmental conditions for each species across its distribution range in Europe at their preferred depths. In addition, by using the EUSeaMap 2023, the total distribution area was filtered by the bottom type that constitutes the known habitat of each species. In this way, a proxy of the total potential habitat surface in km^2^ was calculated for all gastropod species.

From the estimated sizes and the mean temperatures representative of each specific distribution, two relevant life history traits were derived: developmental time and fecundity. Developmental time (DT) was inferred from the model presented by (Gillooly et al. 2002) as:

(1) $$\mathrm{DT}=\left(4/a_{0}\right)*e^{E/\mathrm{kT}}*{\left(\mathrm{\Delta S}\right)}^{\alpha }$$

where(4/a_0_) is a constant, *E* is the activation energy (0.65 eV approx.), *k* stands for the Boltzmann constant, T is temperature in Kelvin degrees, *α* is the allometric exponent (0.25) and ΔS is the difference in size between the initial stages and the juvenile in mm^3^. A second bibliographic compilation (Table S1 in Appendix S2) was used to gather DT data obtained at known temperatures for gastropod species worldwide to perform a calibration where the value of the constant 4/a_0_ could be inferred, thus enabling the calculation of DT for the targeted European species.

Fecundity (*C*) was obtained using the model described by Bueno and López‐Urrutia (2012):

(2) $$\ln\left(\mathrm{Cx}\right)=-0.99*\ln\left(m_{0}\right)+19.15$$

where *Cx* is fecundity per gram of female per day and corrected by temperature and m_0_ is the mass of the initial embryonic stage calculated from IS assuming a density of 1 g/L. This model yielded an extremely good fit (*R* ^2^ = 0.98) for a wide range of animals (Bueno and López‐Urrutia 2012). Accordingly, the number of offspring per an entire female per day at a representative environmental temperature is:

(3) $$C=\mathrm{Cx}\;*M^{0.75}\;*\;e^{–E/\mathrm{kT}}$$

where *M* is the mass of the adult obtained from AS with the same density.

Log transformed sizes, their ratios, DT and *C* were all submitted to analyses of variance (ANOVAs) with mode of development as a factor with 3 levels (non‐pelagic, pelagic planktotrophic and pelagic lecithotrophic). Sums of squares of type III were used in these ANOVAs to account for the imbalance in the number of species within each developmental mode. Kolmogorov Smirnov and Levene's tests were used to check the assumptions of normality and homogeneity of variances prior to ANOVAs. For those variables showing significant differences between developmental modes, Tukey post hoc pairwise comparisons were carried out.

### 2.5 Taxonomic Tree

A taxonomic tree was built for all the European species in the gastropod dataset. The tree was built in R using the as.phylo function from the package ape that produces a classification based on taxonomic hierarchical ranks (Chamberlain et al. 2020). It was built from a species classification based on the World Register of Marine Species (WoRMS, https://www.marinespecies.org/). Libraries ape and taxize were employed to represent and visualise the tree (Paradis and Schliep 2019; Chamberlain et al. 2020). As this tree does not contain real evolutionary distances, it cannot be used in any statistical procedure to correct the effect of phylogeny on the evolution of gastropod life history traits, but it provides a useful overlook of the diversity of the taxa used in this work.

### 2.6 Latitudinal Patterns in Developmental Modes and SAR Models

To obtain distribution maps for the total number of taxa and the proportion of each type of development all over Europe on a scale of 300 m, the individual species‐specific raster layers were added together for all the species and then separately for each developmental mode (non‐pelagic, lecithotrophic and planktotrophic). Then, the proportions for each one of these 3 modes were split in 5° latitudinal bands from 25° to 70° N to look for any latitudinal trend. Representative estimates of these proportions for every latitudinal band were calculated with 3 different methodologies in ascending order of detail and reliability: (1) a single proportion pooling all the species present within each band; (2) a mean proportion calculated as the average of all pixels within each band; and (3) a mean proportion calculated as the average of only those pixels with a minimum number of species to get reliable proportions (*N*_min_) and within regions that have been well surveyed in search of gastropod species according to an independent geographical database. Standard deviations were also calculated for the means obtained from the 2nd and 3rd methodologies to evaluate variability in proportions within each band. To estimate *N*_min_ using the 3rd approach, we designed a test capable of detecting at least a ±30% deviation (ΔP) from the reference mean proportion (ΔP) with respect to the reference mean proportion of non‐pelagic developers in our dataset (*P*), with a statistical power of 0.8 at a significance level of *p* = 0.05. We assumed the probability of observing a proportion not significantly different from the reference follows a normal distribution. Under this assumption, *N*_min_ was calculated as:

(4) $$N_{\min}={\left(\left(Z_{\alpha }+Z_{\beta }\right)/\mathrm{\Delta P}\right)}^{2}\;*\left(P*\left(1-P\right)\right)$$

where *Z*_α_ and *Z*_β_ are the critical values corresponding to the significance level and the desired power, respectively and (*P* * (1−*P*)) represents the variance, according to the Central Limit Theorem. Subsequently, we applied a permutation test (100 permutations) to all pixels containing more species than N_min_ to evaluate whether the proportion of non‐pelagic developers in each pixel significantly differed from the reference. On the other hand, we employed a geographical database independent of the distributions maps built with our gastropod dataset to assess how well a given area was surveyed in search of gastropods. This database was extracted from the Global Biodiversity Information Facility (GBIF, www.gbif.org) for all living taxa within the class Gastropoda in the depth range from 0 to 4000 m for the European seas (25°‐70° N, 25° W‐37° E) and through a timespan of 123 years (1900–2023). A species occurrence matrix was built from it binning individual observations in 0.5° pixels and then the expected number of species per pixel was calculated using the Chao 1 index as:

(5) $$\text{Chao}\;1=S_{\mathrm{obs}}+{f_{1}}^{2}/\left(2*f_{2}\right)$$

where *S*_obs_ is the number of species observed per pixel, and f_1_ and f_2_ stand for the frequency of species observed once (singletons) and twice (doubletons) in a given pixel, respectively. The greater the expected number of species Chao 1 with respect to *S*_obs_ the worse the sampling at a given pixel. Thus, we considered well surveyed areas as those where the relative Chao 1 index (GBIF_RELCHAO = Chao 1/*S*_obs_) was lower than 1.2, meaning that the expected number of species was less than 20% higher than the number of species observed. These calculations were performed in R with the package vegan and dplyr (Oksanen et al. 2022; Wickham et al. 2022). This GBIF database was also used to obtain the number of gastropod species within each superfamily to be compared to the same metric derived from our own dataset. In this way we can evaluate the taxonomic representativeness of our dataset.

We inspected the variables that could be behind the spatial variations of the proportions for each developmental mode only at the regions where proportions can be trusted following the 3rd methodology. Therefore, 0.5° pixel averages for all environmental and primary productivity variables were calculated. Then, logit transformed proportions were submitted to GLMs with environmental and primary productivity variables as predictors. The best GLMs were selected by their Akaike Information Criteria (AIC) with the function dredge in the R library MuMIn (Barton 2022). Models were inspected and the collinearity among their predictors was checked, considering only those predictor combinations where pairwise Pearson's *R* absolute values were below 0.65. In addition, models showing a variation inflation factor (VIF) greater than 3 (a conservative threshold) were discarded. Then, the best GLMs were used to build Spatial Autoregressive (SAR) models which accounted for the lack of independence between adjacent observations in space by weighting them proportionally to their pairwise distance. This weighting system was performed for all pairwise observations within the corresponding patch size, that is, the distance at which the spatial autocorrelation index Moran's I reaches 0 (Legendre and Fortin 1989; Legendre and Legendre 1998). Moran's I spatial correlograms were built in R with the package ncf (Bjornstad 2022) and their x‐intercepts were assumed to be a good proxy of the patch size. At distances greater than the patch size, autocorrelation effects are assumed to be negligible so no weights were applied. SAR models incorporated a parameter that captures the effect of the spatial autocorrelation and its statistical significance, ρ, that ranges from 1 (positive autocorrelation) to −1 (negative). Model performances were obtained as *R* ^2^ estimates from linear regression between predicted and observed logit transformed proportions. These models were carried out in R using the libraries spdep and spatialreg (Bivand and Wong 2018; Bivand et al. 2021).

### 2.7 Multivariate Analyses

To explore how the different life history traits were associated between each other and how they related to environmental variables, multivariate analyses were performed. First, morphological measurements, growth ratios, DT and *C* were all log transformed and scaled, while developmental mode and food type were considered as dummy variables to make a Principal Component Analysis (PCA). Variability explained by the two main principal components PC1 and PC2 was obtained. Then, the loadings for PC1 and PC2 were submitted to Generalised Linear Mixed‐Effects Models (GLMERs) as dependent variables with the taxa‐specific means of environmental and primary productivity variables as predictors and superfamily as a random factor to account for potential phylogenetic effects. The best GLMERs were selected by their AIC with the function dredge in the R library MuMIn (Barton 2022). Models and their VIFs were inspected to prevent excessive multicollinearity, in addition to applying the Pearson's *R* threshold of 0.65. *R* ^2^ was calculated as a measurement of variability explained by the models, with and without the factor superfamily to account for its statistical significance through a Likelihood Ratio Test (LRT). R packages vegan, ape, usdm and tidyr were also used for these analyses (Naimi et al. 2014; Paradis and Schliep 2019; Oksanen et al. 2022; Wickham and Girlich 2022; Revell 2024).

### 2.8 GLMER Binomial Models

In addition to the analyses of the PCA axes as proxies for all the variability in life history traits, we focussed on developmental mode as it is the main life history trait of interest in this study. Therefore we applied binomial GLMERs with pelagic and non‐pelagic development as a binary dependent variable, taxa‐specific environmental and primary productivity variables as predictors and superfamily as a random factor to account for potential phylogenetic effects. Because of the unbalanced number of species with the two modes of development, normalised taxa specific weights were added in the GLMERs. Again we used the dredge function, the Pearson's *R* criteria and the VIF to select the best model. To infer the statistical significance of the phylogeny, the best GLMER model was compared to a GLM with the same environmental predictors but without the superfamily factor through a LRT. Overall *R* ^2^ was used to evaluate the goodness of fit of the model together with its accuracy for all species but also for each mode of development.

## 3 Results

### 3.1 Hydrography

**FIGURE 1:**
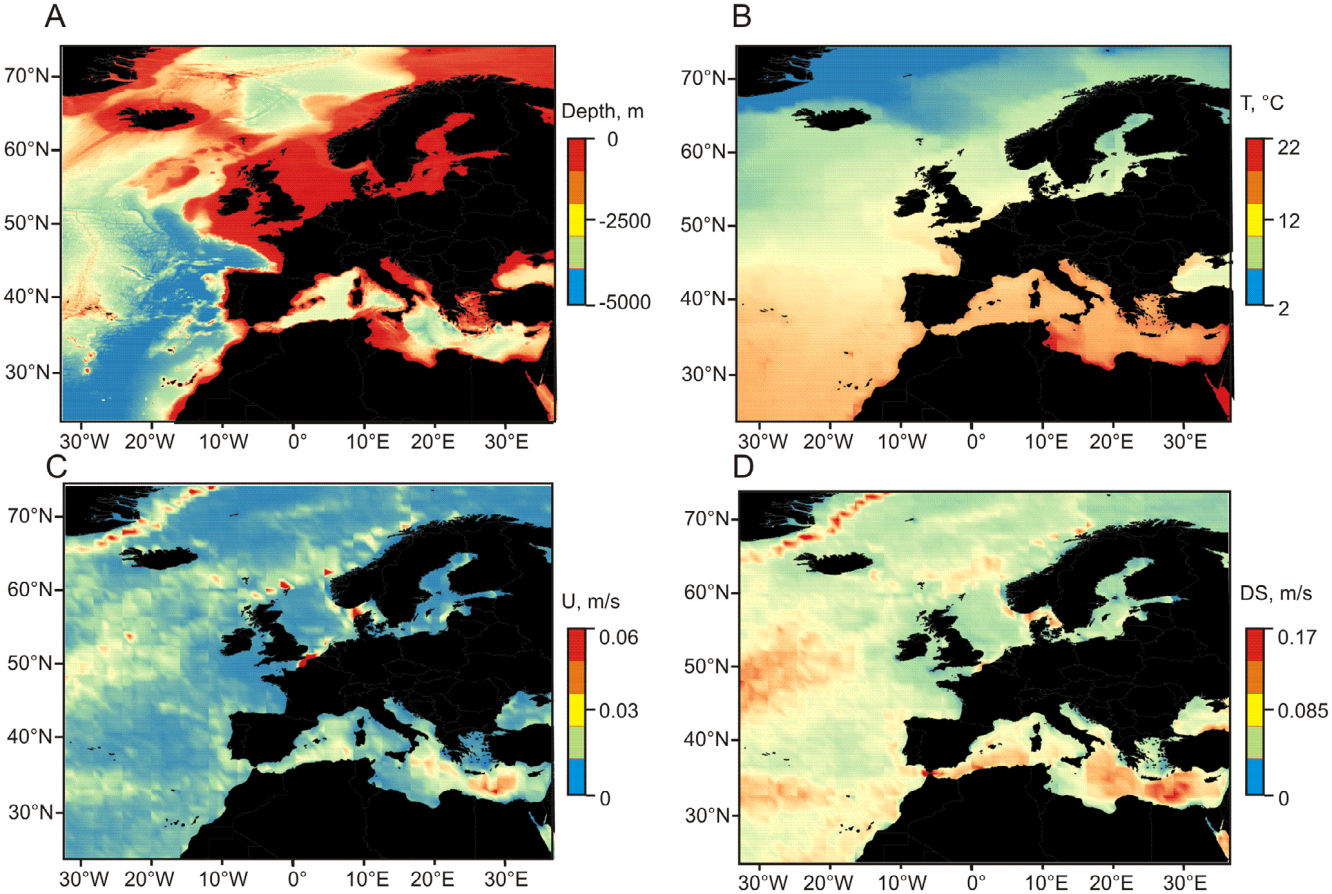
Spatial distribution of hydrographical variables in European waters. (A) Depth, (B) water column mean temperature (T), (C) mean current (U), and (D) dynamic stratification.

The GEBCO topographic dataset allowed a good representation of bottom depth for all European seas (Figure 1A). On the other hand, the NRL HYCOM+NCODA provided estimates of temperature and currents in agreement with the main hydrographical patterns in the region. Water column temperatures peaked in the Eastern Mediterranean and along the coasts of Tunisia (22°C). As expected, the thermal latitudinal gradient along the Atlantic coast is milder (17°C off Morocco to 7°C off Northern Norway) compared to that found further West along the North Atlantic (17°C off Madeira to only 2°C off Southern Greenland, Figure 1B). Therefore, the model captures the influence of the Atlantic Current and its heat transport towards the European Atlantic shores. Regarding overall circulation, U ranges from several mm/s to 6 cm/s along the shelfbreaks off Greenland and Scotland‐Southern Norway, and also at two of the narrowest straits in the region, the English Channel and the Skagerrak (Figure 1C). Dynamic stratification reached vertical standard deviations around 17 cm/s at the same straits and also at the entrance of the Mediterranean Sea, and remains high along the coasts of Algeria, the Eastern Mediterranean and in the mid‐Atlantic at two hotspots centered around 35° and 45° N (Figure 1D).

### 3.2 Primary Productivity Variables

**FIGURE 2:**
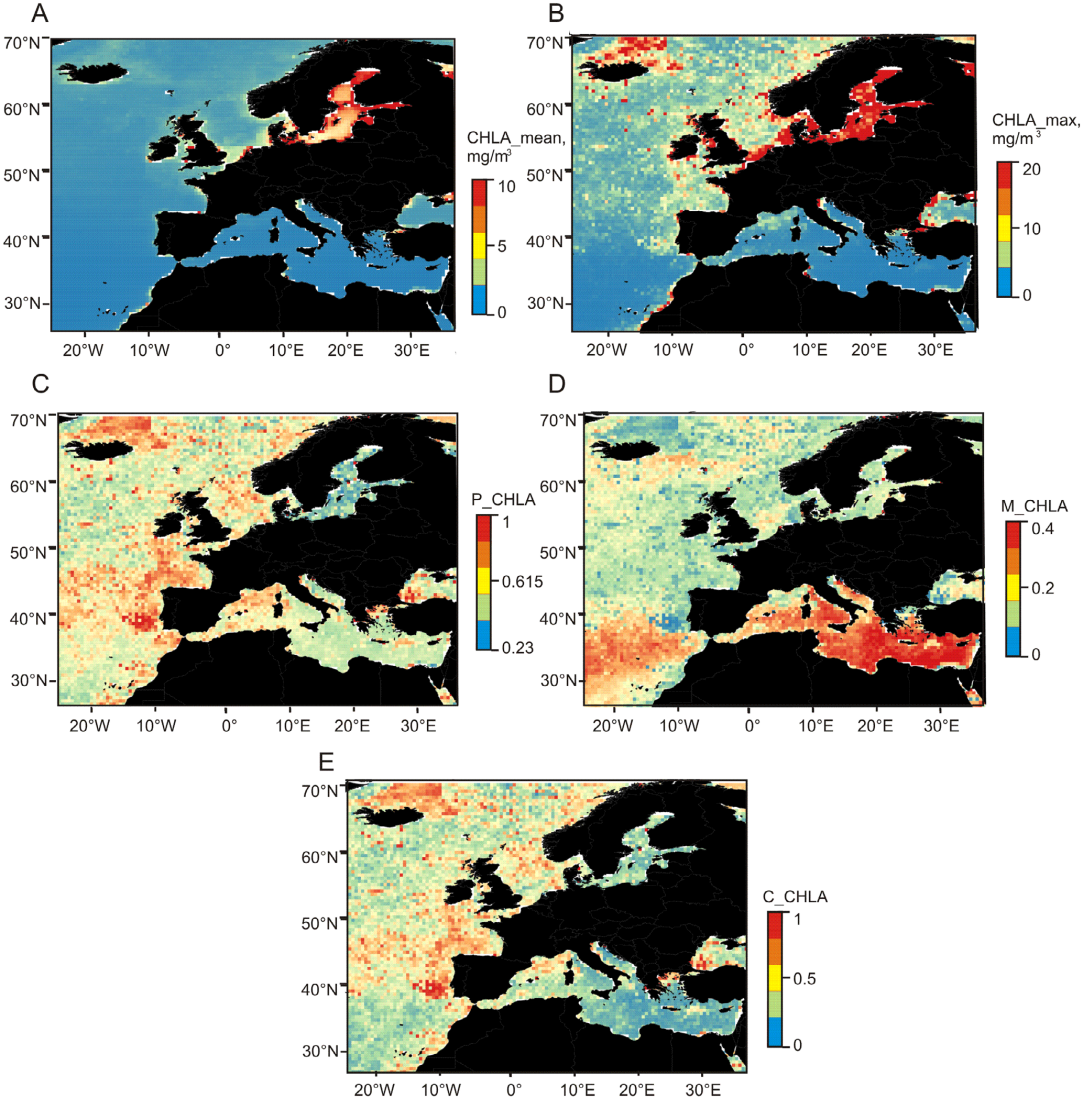
Spatial distribution of primary productivity variables in European waters. (A) surface mean chlorophyll‐a (CHLA_mean), (B) surface maximum chlorophyll‐a (CHLA_max), (C) overall predictability (P_CHLA), (D) contingency (M_CHLA), and (E) constancy (C_CHLA).

The 2003–2022 chlorophyll‐a dataset from the satellite Aqua MODIS successfully recreated the spatial patterns in productivity in European seas. CHLA_mean reached maximum values of 10 mg/m^3^ only along the coasts of the Baltic Sea, but CHLA_max presented higher values up to 20 mg/m^3^ at many other regions: offshore Baltic Sea, North Sea, English Channel, Irish Sea, Northern coasts of Iceland, and some coastal hotspots off Morocco, Northern Spain, Portugal, Italy, Tunisia and Northern/Western Black Sea (Figure 2A,B). Colwell's indexes based on the chlorophyll‐a time series revealed some contrasting patterns. Overall predictability P_CHLA was high (0.7) along the Atlantic coasts from Southern Portugal to Ireland, including the Gulf of Biscay, and to the north of Iceland, while it reached its minimum (0.23) in the Baltic Sea (Figure 2C). Contingency M_CHLA reached values up to 0.4 within a latitudinal band between 30° and 40° N that includes the Atlantic and the Mediterranean Sea, especially off Greece and Turkey, and presented minimum values around 0 off Southern Portugal and off Northern Iceland (Figure 2D). Spatial patterns in constancy C_CHLA mirrored the distribution of P_CHLA with maxima close to 0.7 off Portugal, Bay of Biscay and Northern Iceland and minima around 0 in the Eastern Mediterranean and Baltic seas (Figure 2E).

### 3.3 Gastropod Dataset

**FIGURE 3:**
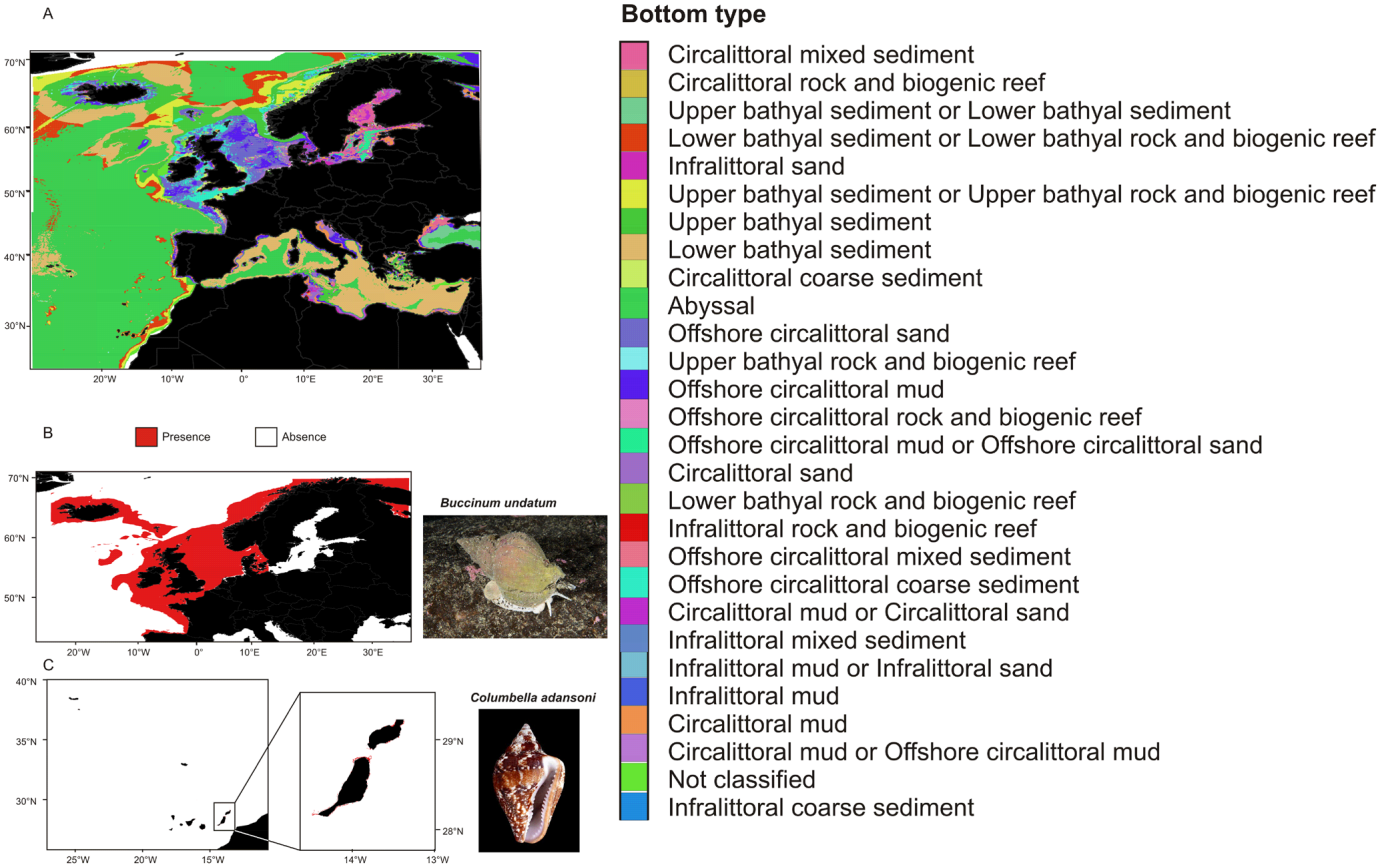
(A) EUSEAMAP bottom habitats and distributions for two particular examples of (B) the most widespread species, *Buccinum undatum* (2,019,150 km^2^, 5–600 m depth, all kinds of bottom habitats), and (C) *Columbella adansoni* (231 km^2^, 0–15 m depth, rocky, sandy and muddy bottoms) with an additional inset to better show the coastal distribution of these species along the coasts. Shell pictures were made by Bernard Picton and H. Zell and shared under licenses that allow reuse (Creative Commons Attribution 4.0 International and Creative Commons Attribution‐Share Alike 3.0 Unported).

Detailed information of habitat, depth and life history traits was found for 94 species of gastropods. Regarding developmental mode, 33 species were non‐pelagic, 51 were planktotrophic and 10 were lecithotrophic. On the other hand, 51 species were carnivorous, 4 were omnivorous and 39 were herbivorous. Depth ranges from a maximum of 3006 m for *Colus islandicus* to the intertidal for many different species. Bottom habitat was predominantly rocky for 53 species, muddy for 19, sediment of variable grain size for 7, sandy for 4 and a mixed combination of these categories for other 11 species. When these taxa specific habitats were taken into account through the EMODnet Seabed habitats raster layer in conjunction with depth, potential habitat surfaces were obtained from each species. This metric ranged from 2,019,150 km^2^ in *Buccinum undatum* to only 231 km^2^ in *Columbella adansonii* (Figure 3).

**FIGURE 4:**
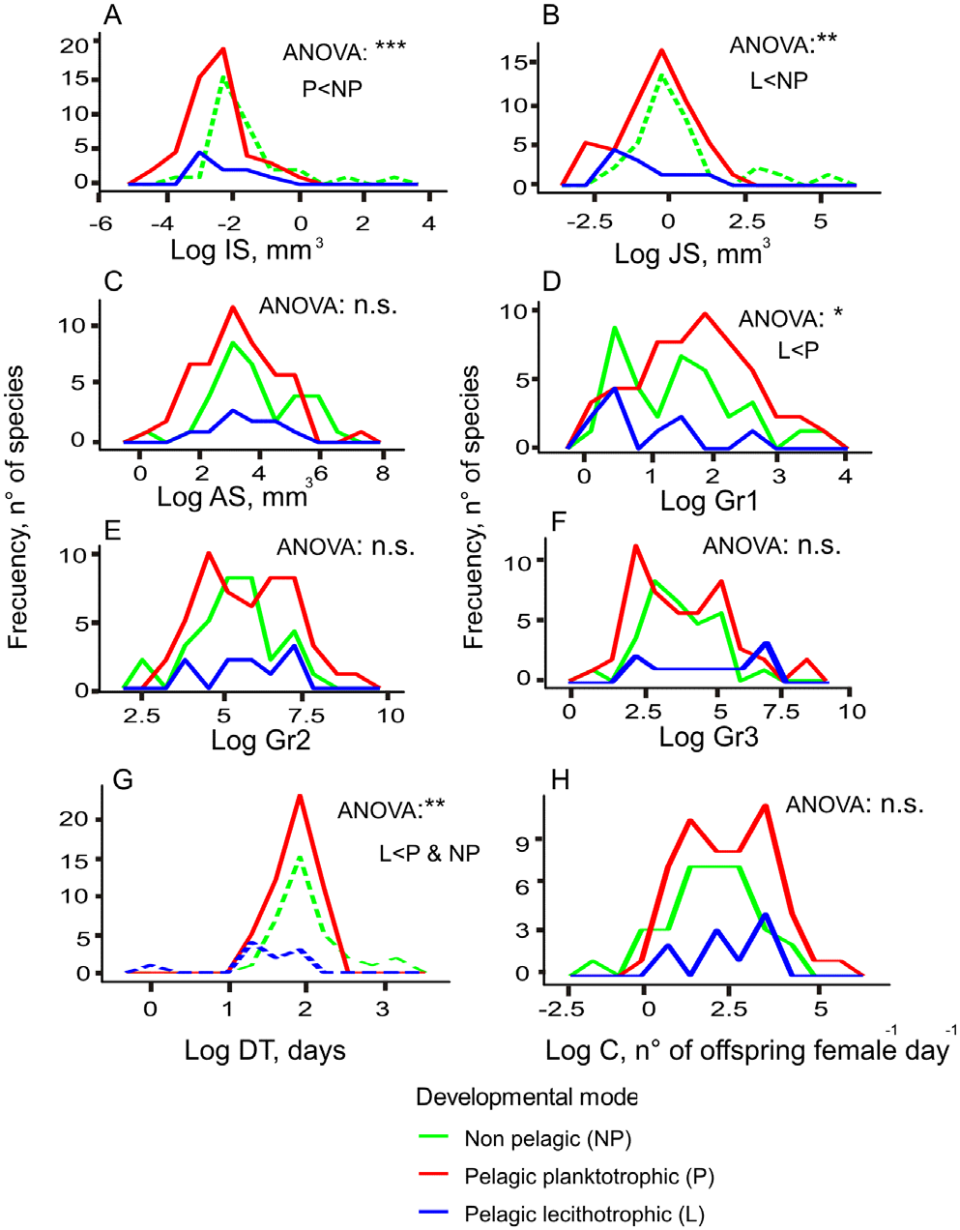
Frequency plots for growth‐related variables (A) initial size (IS), (B) juvenile size (JS), (C) adult size (AS), (D) ratio between juvenile and initial sizes (Gr1), (E) ratio between adult and juvenile sizes (Gr2), (F) ratio between adult and initial sizes (Gr3), (G) development time (DT), and (H) fecundity (C). Discontinuous lines show distributions that are not normal according to KS tests. The significance of the ANOVAs is represented (0.05 < *p*; 0.01 < *p* < 0.05: *; 0.001 < *p* < 0.01: **; *p* < 0.001: ***). Significant pairwise comparisons from the Tukey post hoc tests are also indicated with letters.

Morphological characteristics and life history traits varied widely between species depending on their developmental mode (Figure 4). Initial and juvenile sizes were significantly bigger in non‐pelagic species than in planktotrophic and lecithotrophic species, respectively (Figure 4A,B). Although these patterns have to be taken with caution due to the lack of normality in the distribution of non‐pelagic developers, only they overpassed 100 and 1000 mm^3^ in initial and juvenile sizes, respectively. Juvenile sizes were not constant and positively correlated with initial sizes. On the other hand, adult sizes did not differ between developmental modes (Figure 4C). Regarding growth ratios, Gr1 was significantly greater in planktotrophic larvae compared to lecithotrophic, as their juveniles were usually around 100 times bigger than the initial larvae, 1–2 orders of magnitude bigger than Gr1 modes for lecithotrophic and non‐pelagic taxa (Figure 4D). Differences in Gr2 and Gr3 between developmental modes were not significant, although Gr2 only reached ratios between adult and juvenile sizes of 1*10EXP8 in the case of planktotrophic species (Figure 4D,E). Developmental times DT were calibrated using the database for 79 species worldwide (Table S1 in Appendix S2). Eventually, we obtained a very significant linear fit between DT and the temperature‐size metabolic term that explained up to 57% of the variability in DT. We then consider the slope of this fit (5.796EXP‐10 days/mm^0.75^) as a good proxy of the constant 4/a_0_ and used it to obtain DT estimates for our European taxa following Equation (1) (Figure S1 in Appendix S2). DT most frequent values were around 100 days for non‐pelagic and planktotrophic species, significantly greater than those for lecithotrophic larvae in the order of tens of days (Figure 4G). Again, the significance of this result has to be taken with caution due to the lack of normality in the distributions of non‐pelagic and lecithotrophic developers. Fecundity estimates *C* did not differ significantly among developmental modes. Anyway, they usually ranged between 10 and 1000 individuals day^−1^ female^−1^ in the case of non‐pelagic species, while for larval forms the modes were located around 10,000 individuals day^−1^ female^−1^. Only some planktotrophic species produced more than 100,000 individuals day^−1^ female^−1^ (Figure 4H).

### 3.4 Phylogenetic Tree

**FIGURE 5:**
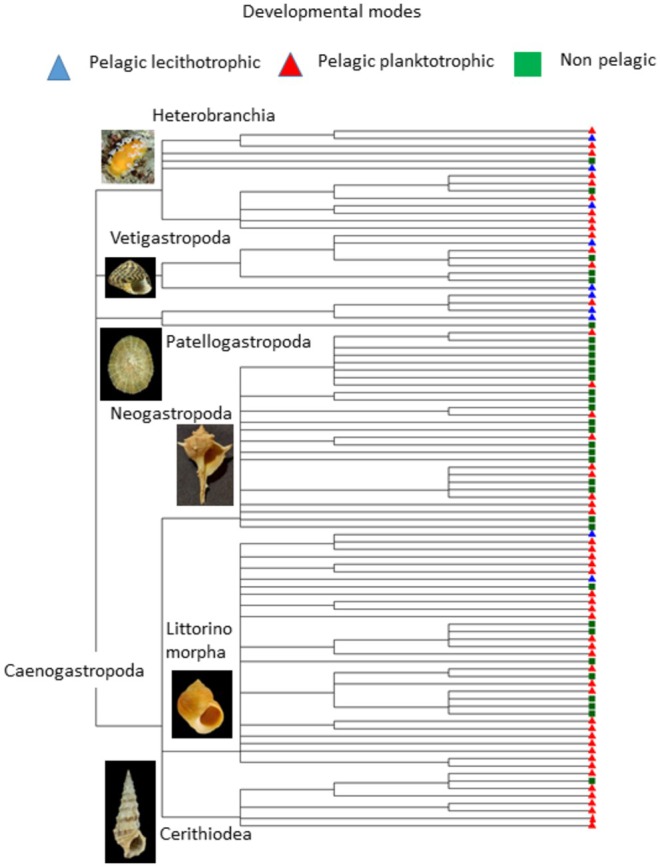
Taxonomic tree for the 94 gastropod species analysed in this study. Shell pictures were made by Anton Chichvarkhin, Marcus Stigwan and H. Zell and shared under licenses that allow reuse (Creative Commons Attribution 4.0 International and Creative Commons Attribution‐Share Alike 3.0 Unported).

Our gastropod dataset covered the full taxonomic complexity of this mollusc class in Europe. The 94 species analysed were distributed in 45 different families and 69 genera across the 4 main subclasses Caenograstropoda, Heterobranchia, Patellogastropoda and Vetigastropoda (Figure 5). The three modes of development were widespread across the whole phylogenetic tree and there was not an evident association between any given taxonomic group and any given developmental mode (Figure 5).

### 3.5 Latitudinal Patterns in Developmental Modes and SAR Models

**FIGURE 6:**
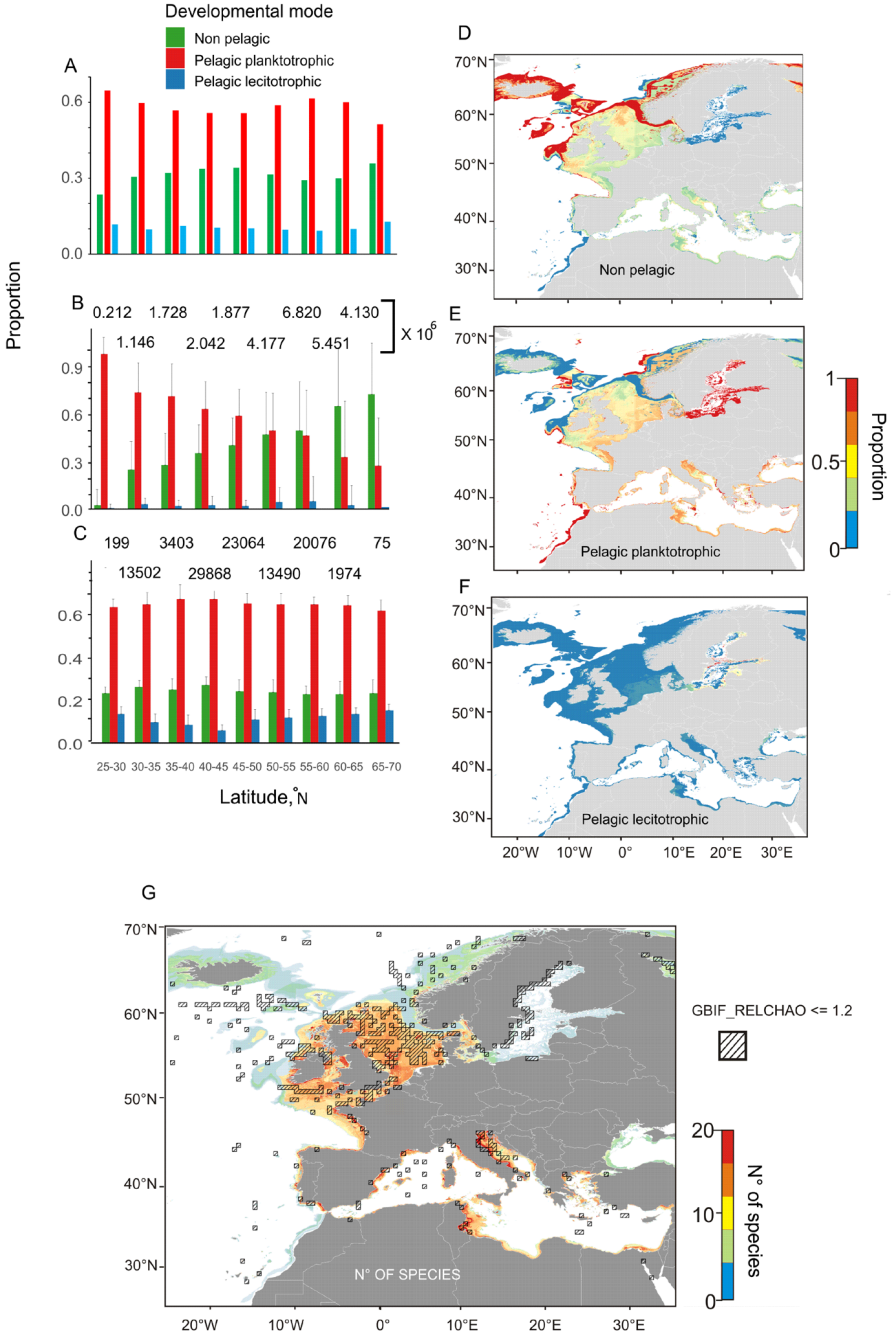
(A) Bar graph showing the proportion of species belonging to the three developmental modes within 5° latitudinal bands from 25° to 70°N. The number of species at each latitudinal band from south to north is 34, 72, 81, 86, 88, 73, 65, 60 and 39, respectively. (B) Same as (A), but with the mean proportions obtained through averaging over all 444 m pixels within each latitudinal band. Dark bars stand for standard errors. The number of pixels is shown above each latitudinal category. (C) Same as (B), but with the mean proportions obtained through averaging over all 444 m pixels with a relative GBIF Chao 1 index smaller than 1.2 and with more than 20 species according to our distribution model. (D, E and F) show the distribution of the proportion of species with non‐pelagic, pelagic planktotrophic and pelagic lecithotrophic development, respectively. (G) Distribution of the number of species of gastropods according to our model. A superimposed line pattern shows areas where GBIF relative Chao 1 index is smaller than 1.2.

Taxa‐specific distribution maps were pooled by developmental mode to obtain the proportion of each mode with a spatial resolution of 300 m and then used to extract latitudinal patterns using 5° latitude bands. When we applied this procedure just attending to the presence of each developmental mode within each band, then there was not any latitudinal pattern consistent with Thorson's rule (Figure 6A). The prevailing mode was pelagic planktotrophic at all bands, attaining 50%–65% of the species present at each latitude, followed by pelagic lecithotrophic (around 30%) and non‐pelagic (around 10%). However, if the 300 m pixel average proportion for all pixels within each band is considered instead of just the presence/absence procedure, then a clear latitudinal trend matching Thorson's rule appeared (Figure 6B). Non‐pelagic species presented a conspicuous northwards rise in proportions from 2% to 65%, while pelagic planktotrophic taxa followed the opposite pattern (from 95% to 25%). Proportions for lecithotrophic species were always too low irrespective of latitude (around 5%) to infer any trend. As all pixels within each band were considered, the number of pixels to perform the calculations was always in the order of millions and standard deviations were substantial (up to 30%, especially for non‐pelagic species in the north). Finally, when we considered only reliable proportions at pixels with more than *N*_min_ = 20 species within relatively well surveyed areas according to GBIF (GBIF_RELCHAO < 1.2), any latitudinal trend disappeared completely (Figure 6C). Planktotrophic development was again the prevailing mode at all latitudes with mean proportions around 65%, while the non‐pelagic and lecithotrophic modes only attained proportions of 25% and 10%, respectively. The number of pixels available for these calculations was largely reduced to the order of thousands to tens of thousands and even further at the northernmost and southernmost bands (75 and 199 pixels, respectively). Standard deviations were also much smaller, always below 10%. On the other hand, when the proportions of species belonging to each superfamily were compared in between GBIF and our dataset, maximum deviations never exceeded 6%. Therefore, our dataset presents a relatively good taxonomic coverage.

These different patterns arising from the 3 different methodologies largely matched the proportion maps for each mode of development (Figure 6D–F) and the restrictions imposed by *N*_min_ and GBIF_RELCHAO (Figure 6G). Proportions higher than 80% were reached for the non‐pelagic mode to the northeast of Scotland, Iceland and Southern Greenland, but along the coasts of Norway they remained below 30% (Figure 6D). To the south proportions fell below 30%, with some hotspots off Northwestern Iberia and the Adriatic sea reaching 50%. Minimum proportions (close to 0) were observed along the coasts of Morocco and in the Baltic Sea. The opposite distribution pattern was followed by the proportions of planktotrophic species (Figure 6E) with a general negative northward trend that disappeared off Norway and in the Baltic Sea, which kept proportions above 75%. Proportions for the lecithotrophic development mode were much lower all over Europe (Figure 6F), with some exceptions in the North Sea (up to 20%) and especially in the Baltic Sea (up to 75%). When we inspected the number of species per pixel obtained from our distribution maps (Figure 6G), it reached relatively high values between 10 and 20 species in the North Sea and at some hotspots within the Mediterranean Sea off Spain, Italy and Tunisia. In fact, values above the *N*_min_ threshold (20 species) were only found at these regions and other very coastal areas. Twenty species was the result obtained for *N*_min_ given a reference mean proportion of non‐pelagic developers of 0.351 (33 out of 94 species), an ΔP of ±30% and a statistical power of 0.8 at a significance level of *p* = 0.05. Permutation tests revealed that there were no significant deviations from the reference proportion of 0.351 for any pixel with a number of species equal to or above *N*_min_. On the other hand, the well‐surveyed areas where GBIF_RELCHAO was below 1.2 were found around the British Islands, the North Sea, the Mediterranean regions off Spain, Italy and Tunisia, some very coastal locations elsewhere (especially in the Baltic Sea) and some offshore spots in the middle of the North Atlantic. Regardless of the relatively good overlap between these regions and those where *N*_min_ was overpassed, they both were a reduced fraction of the total surface considered in our initial distribution maps.

**FIGURE 7:**
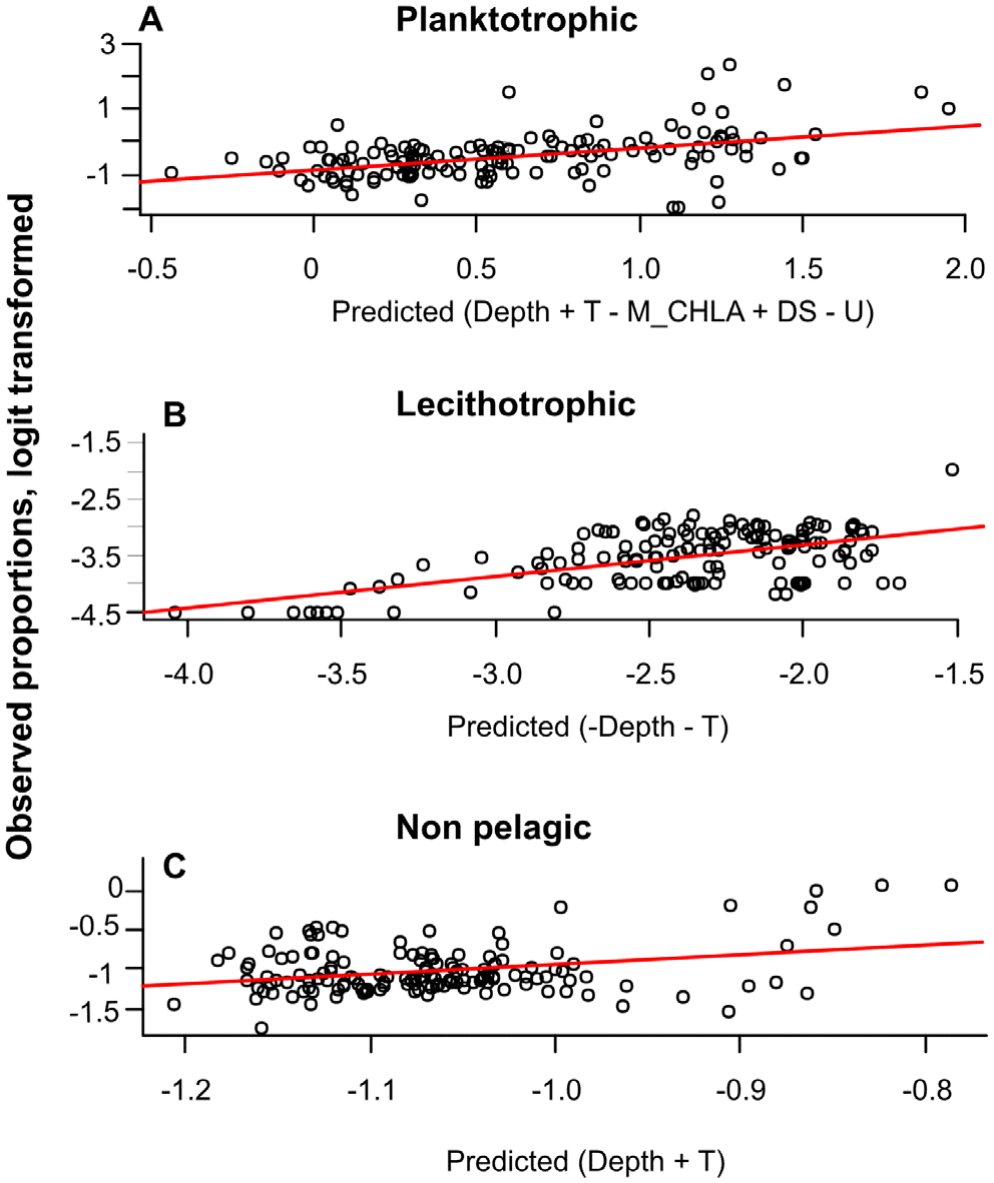
Observed versus predicted plots from the SAR models for logit transformed proportions of planktotrophic (A), lecithotrophic (B) and non‐pelagic (C) modes of development per 0.5° pixels within well surveyed regions (relative GBIF Chao 1 index smaller than 1.2) with more than 20 species according to our distribution maps. The environmental variables that constitute the best model are shown in the x‐axis with their corresponding sign.

The best SAR models explained moderate amounts of variability in developmental mode proportions within regions complying with the *N*_min_ and GBIF_RELCHAO thresholds (in between 7% and 41%, Table 1, Figure 7). Spatial autocorrelation effects were only significant for the planktotrophic mode, with a Moran's I patch size of 841 km. In fact, this was the only model where the explicit inclusion of the spatial autocorrelation significantly improved the fit according to AIC values (Table 1). Increments in the proportions of planktotrophic species were associated with greater depths, higher temperatures, reduced chlorophyll‐a seasonality (lower M_CHLA values, although this variable was not significant) and weaker and more vertically stratified currents. On the other hand, lecithotrophic taxa higher proportions correlated with shallower depths and colder temperatures, while just the opposite occurred for non‐pelagic developers (Table 1, Figure 7).

**TABLE 1:** Results of the SAR models performed on the logit of the proportion of gastropod species with planktotrophic, lecithotrophic or non‐pelagic development per 0.5*0.5° pixel.

Dependent variable	Predictors	Estimate	Std. error	*z* value	Pr(>|z|)	Rho	Moran's I patch size, km	*R* ^2^	AIC
Planktotrophic	**(Intercept)**	**−2.0229306**	**0.5098452**	**−3.9677**	**7.26E‐05**	−1	841	0.2221	413.25
	**Depth**	**0.0118702**	**0.0038427**	**−3.089**	**0.002008**	**		***	*418.4*
	**T**	**0.160255**	**0.0396077**	**4.0461**	**5.21E‐05**				
	M_CHLA	−2.0589061	1.1589732	−1.7765	0.0756519				
	**DS**	**20.0821066**	**5.346123**	**3.7564**	**0.0001724**				
	**U**	**−17.572179**	**7.712438**	**−2.2784**	**0.0227015**				
Lecithotrophic	**(Intercept)**	**−1.0829219**	**0.5207257**	**−2.0796**	**0.03756**	−0.33	431	0.4142	246.96
	**Depth**	**−0.0212019**	**0.0022116**	**9.5867**	**< 2.2e‐16**	n.s		***	*246.81*
	**T**	**−0.102756**	**0.0182857**	**−5.6195**	**1.92E‐08**				
Non‐pelagic	**(Intercept)**	**−1.4354219**	**0.3307056**	**−4.3405**	**1.42E‐05**	−0.039	474	0.0782	27.99
	**Depth**	**0.0036364**	**0.001022**	**−3.5583**	**0.0003733**	n.s		***	*26.01*
	**T**	**0.0153908**	**0.0076939**	**2.0004**	**0.0454571**				

*Note:* Significant terms in the models are shown in bold. Significance levels (*p* < 0.001) for the whole models and their spatial autocorrelation fit are shown with three asterisks in the Rho and *R* ^2^ columns, respectively. The AIC values of the linear models without the spatial autocorrelation fit are reported in italics.

### 3.6 Multivariate Analyses

**FIGURE 8:**
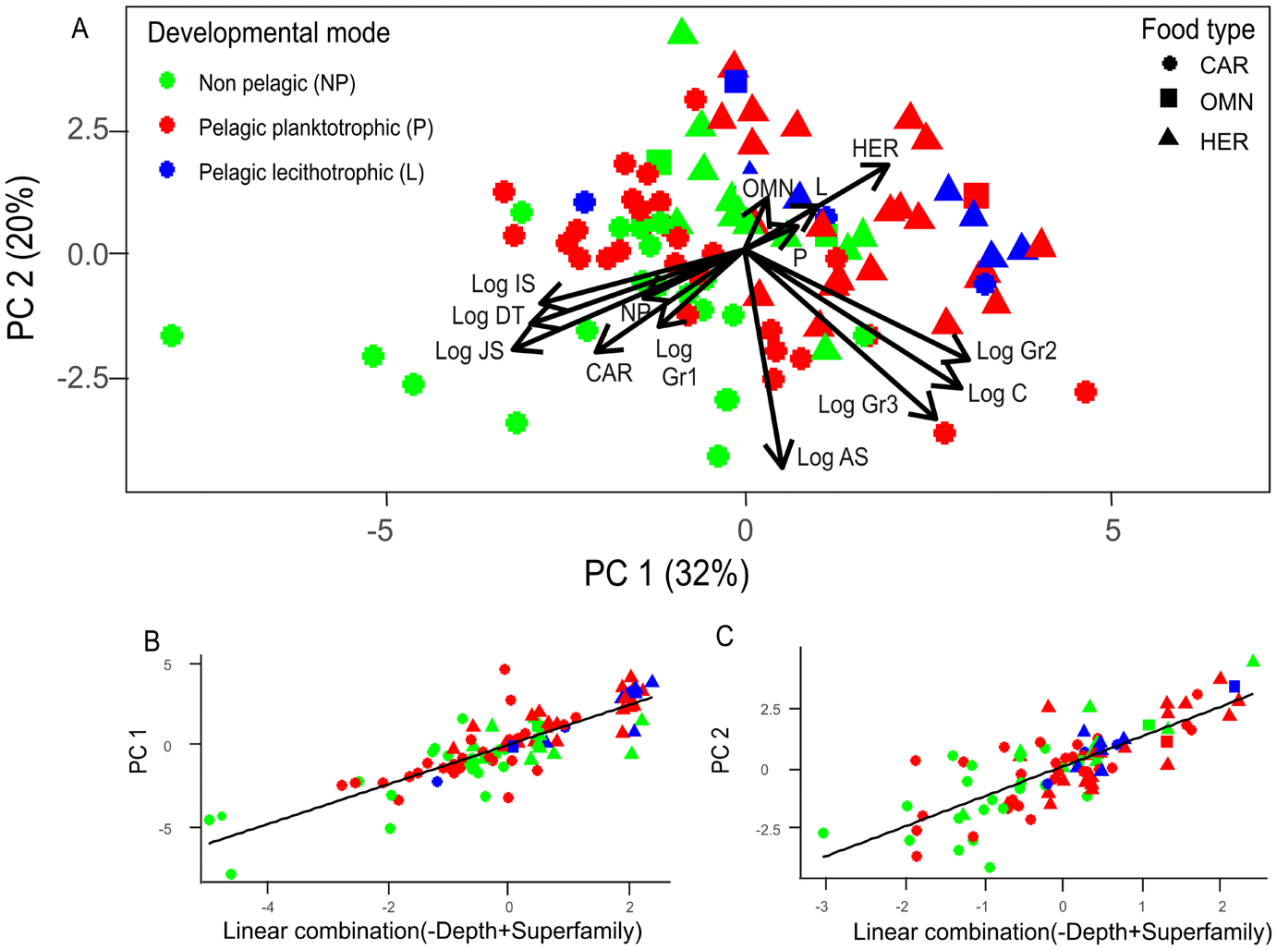
(A) PCA plot for life history traits in 94 species of European gastropods. Symbol shapes and colours stand for food type and developmental mode, respectively. See Material and Methods section 2.4 for the acronyms of each life history trait. Abbreviations CAR, OMN and HER stand for carnivorous, omnivorous and herbivorous, respectively. (B) Predicted versus observed plot of the best GLMER and LRT tests for PC1. The environmental variables that constitute the best model are shown in the *x*‐axis with their corresponding sign. (C) Same as B but for PC2.

The PCA statistical procedure revealed conspicuous associations between the different morphological and life history traits in gastropod species (Figure 8A). The two main axis PC1 and PC2 explained up to 52% of the total variability in traits between the different taxa and successfully separated them by their developmental mode, especially following the binomial pelagic/non‐pelagic division. Pelagic modes of development were associated with shorter developmental times, smaller initial and juvenile sizes and their ratio Gr1 and herbivorous/omnivorous feeding habits, while the opposite held for non‐pelagic species. Gr2 and Gr3 were more linked to fecundity and adult size. The GLMER models showed increments in PC1 with shallower depths and a significant effect of the superfamily factor accounting for phylogenetic effects (Table 2, Figure 8B). The model explained up to 68% of the variability in PC1 loadings, which are in turn higher for pelagic developers in general (Table 2, Figure 8B). PC2 also increased with shallower depths and was significantly influenced by the phylogeny, according to the best model that explained up to 62% of the variability in PC2 loadings with the inclusion of the superfamily factor. For both PCs, superfamily explained more than 50% of total variability. Like for PC1, PC2 loadings were higher in pelagic developers (Table 2, Figure 8C).

**TABLE 2:** Results of the GLMER and LRT tests for the life history traits PCA axes.

Model	Variable	Estimate	Std. err	*t*‐value	*p*	*R* ^2^	Overall *R* ^2^	AIC
PC1	(Intercept)	0.02141	0.36644	0.058	1		0.6839	379.87
	**Depth**	−0.59397	0.16052	−3.7	**7.85E‐05**	**0.1567**		*396.02*
	**Superfamily**				**2.05E‐05**	**0.5960**		
PC2	(Intercept)	0.2496	0.2723	0.917	1		0.6215	341.25
	**Depth**	−0.3326	0.1351	−2.462	**0.004812**	**0.0832**		*358.18*
	**Superfamily**				**1.36E‐05**	**0.5088**		

*Note:* Significant terms in the model are shown in bold. The AIC of the linear model without the phylogenetic effect is reported in italics.

### 3.7 Binomial GLMER Models

**FIGURE 9:**
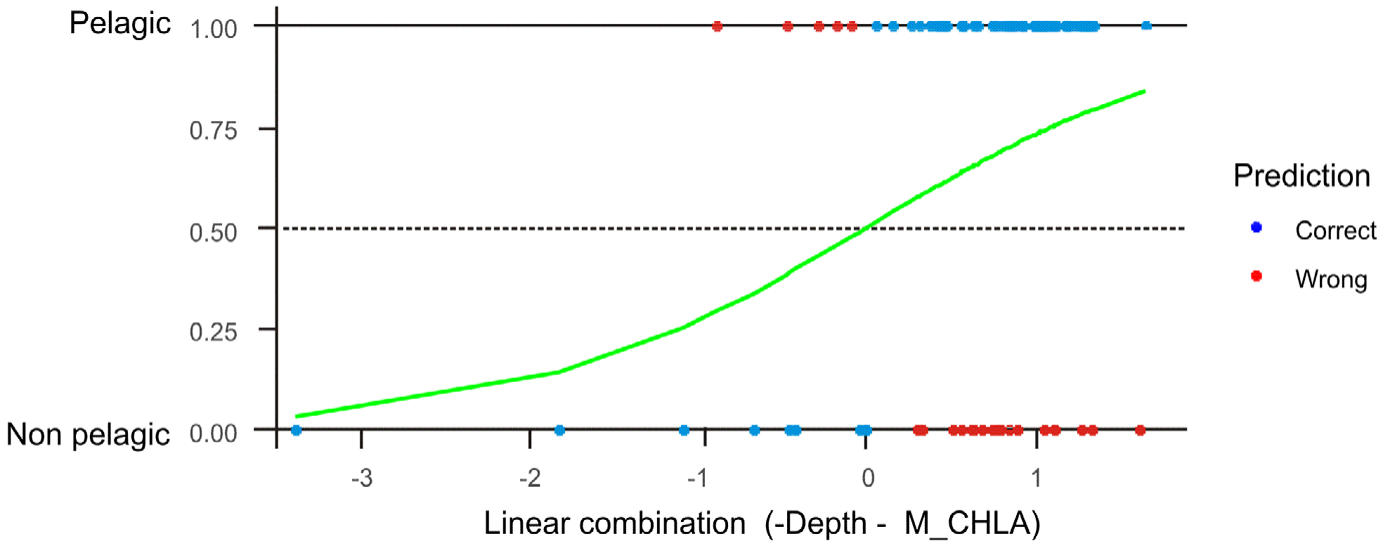
Plot of the probability of pelagic development (*y*‐axis) as a function of the linear combination of depth and chlorophyll seasonality (*x*‐axis) according to the best binomial GLMER models (green line). Individual points represent the known developmental mode of the 94 species either as pelagic (located in the upper horizontal line) or non‐pelagic (on the *x*‐axis). Blue and red points stand for correct and wrong developmental mode assignations, respectively, following model predictions.

The best binomial GLMER model for the probability of a given species to present a non‐pelagic (0) or pelagic (1) mode of development included just depth and chlorophyll‐a seasonality as environmental predictors (Table 3, Figure 9). The probability of becoming a pelagic developer decreased with increasing depths (although its effect was not significant) and M_CHLA values. According to the LRT between the GLMER model with the superfamily factor and the GLM without it, the role of the phylogeny in determining the developmental mode was negligible (Table 3). Actually, AIC estimates pointed out that the model showed a slightly better fit without including any phylogenetic effect. Overall accuracy was 69%, reaching a 91% for pelagic species and just 27% for non‐pelagic ones, with 14% in *R* ^2^. The model output was the same regardless of the use of taxa specific weights to correct the slight imbalance in the number of species within each category (61 vs. 33).

**TABLE 3:** Results of the binomial GLMER and LRT tests performed on the developmental mode categories pelagic and non‐pelagic for 94 species of European gastropods.

Predictors	Estimate	Std. error	*z* value	Pr(>|z|)	*R* ^2^	AIC	logLik	Overall *R* ^2^	Accuracy
**(Intercept)**	**3.432693**	**1.276884**	**2.6883**	**0.0071**		120.79	−56.4	0.1411	69% total
Depth	−0.00471	0.002415	1.9496	0.051	0.0546	*118.79*			91% pelagic
**M_CHLA**	**−14.19282**	**6.912224**	**−2.0532**	**0.04**	**0.0394**				27% non‐pelagic
Superfamily				1	3.50E‐15				

*Note:* Significant terms in the model are shown in bold. The AIC of the linear model without the phylogenetic effect is reported in italics.

## 4 Discussion

In this work we have taken advantage of the last advancements in remote sensing, modelling and analytical procedures to test the validity of an old marine biogeographical paradigm in the context of modern macro‐ecology. The detailed spatial, multivariate and phylogenetic analyses performed on 94 European marine gastropods have revealed that Thorson's rule is probably an artefact produced by prior incomplete and spatially constrained surveys. Instead, our results suggest that both the probability of developing a pelagic larval phase and the proportion of species presenting that phase at a given area are associated with two main environmental drivers that do not necessarily vary with latitude: habitat depth and seasonality of primary productivity. However, phylogeny exerts an important influence when all morphological and life history traits are considered together.

### 4.1 Deceptive Latitudinal Trend

In 1950 Gunnar Thorson presented an apparent northward‐increasing trend in the percentage of prosobranch gastropod species with non‐pelagic development throughout the North Atlantic (Thorson 1950). The regions surveyed in search of gastropods were 7 and followed a rather contorted latitudinal transect: from Eastern Greenland to the southeast through Northern and Southern Iceland, the Faroes Islands, Southern Scandinavia, and then to the southwest towards the English Channel and finally the Canary Islands. Later on, Thorson supported the latitudinal gradient with other observations, and, with time, it became a biogeographical rule (Thorson 1936, 1940, 1950; Mileikovsky 1971). The rule was based on surveys carried out in the 30's and the 40's, that constitute an impressive achievement given the resources available for oceanographic research back then. Still, there was an uneven spatial distribution of sampling effort that allows for great discrepancies in latitudinal trends depending on the selection of the regions to be sampled. For instance, if the Northern Scandinavian shelf, with its relative high presence of planktotrophic species (Figure 6E), was to be surveyed instead of Eastern Greenland as the northernmost location, then the pattern would have been blurred and probably the biogeographical rule would not have been stated. Nevertheless, the few available observations back then made Thorson assume that pelagic development was almost absent over the Arctic and totally suppressed over the Antarctic region, including the shallow continental shelves (Thorson 1950).

Thanks to the development of both biological and physical oceanography during the rest of the 20th century, more complete distributions for many more species of gastropods and other marine species were obtained. As a consequence, a better inspection of latitudinal trends became possible, as all surveyed regions within a given latitudinal range could be taken into account. In this context, poleward gradients for the proportions of pelagic and non‐pelagic gastropods for both the Pacific and the Atlantic coasts of South America using all locations within bands of 5° in latitude were inferred (Gallardo and Penchaszadeh 2001). This procedure was expanded for more taxa in many other regions (Marshall et al. 2012; Pappalardo et al. 2014; Pappalardo and Fernández 2013). Nevertheless, this procedure is over‐representing those rare species that do occur within a given latitudinal band but only in a few specific locations. For instance, percentages of planktotrophic taxa present at latitudes of 50°–55° N and 60°–65° N were fairly similar (Figure 6A), but their spatial distribution is much more constrained within the 60°–65° N band to the coasts of Southern Scandinavia than within the 50°–55° N band, where these species are widespread all over the North and Baltic seas (Figure 6E).

Eventually, in the 21st century better and more extensive samplings were carried out together with a proper characterisation of bottom habitats with products like the EUSeaMap 2023. These resources made high resolution potential distribution maps possible (Andersen et al. 2018; Callery and Grehan 2023) and, in turn, pixel specific proportions could be inferred. Moreover, massive biogeographical databases like GBIF allow for the first time to assess the real sampling efforts for vast areas with a historical perspective. Thus, for European gastropods such assessment reveals that, although huge advancements have been made since the original Thorson's 7 locations, extensive marine areas usually thought to be well studied like the European seas, are still severely undersampled (Figure 6G). The consideration of pixel specific proportions calculated with too few species to rely on such proportions at areas not sufficiently well surveyed led to a latitudinal trend consistent with Thorson's rule (Figure 6B). In essence, extensive deep areas to the Northwestern Atlantic off Iceland and Greenland with very few species, all of them non‐pelagic developers, cause this pattern to emerge (Figure 6D–G). However, most of these areas have not been properly sampled in search of gastropods yet. It can be argued that such northern deep regions may not present more than just the 5–10 gastropod species that our distribution maps suggest (Figure 6G) and therefore the minimum number of 20 species threshold could never be attained there, no matter the sampling effort. Nevertheless, there are more than 50,000 species of marine gastropods in the world (Strong et al. 2008) and recent surveys around Iceland point to a species richness around 200 in these waters (Egilsdottir et al. 2019). Thus, there are more than enough gastropod species to infer reliable proportions in developmental modes even at these northern offshore regions, but we lack the detailed knowledge on their life history traits to include them in the analyses presented here. Lastly, when only those pixels complying with the GBIF and *N*_min_ criteria were analysed, no latitudinal trend was found (Figure 6C). We are aware that this is a very conservative criteria that reduces the number of pixels available for the analysis, especially at the southernmost and northernmost bands, but relaxing these minimum reliability thresholds may produce artificially biased patterns (Figure 6B). We believe that the way to proceed is to progressively increase the areas available for biogeographical analyses of this sort by enhancing sampling effort and research on the life cycles of the organisms inhabiting the new surveyed areas.

### 4.2 Developmental Mode—Phylogeny Decoupling

Spatially explicit proportions of each developmental mode (Figure 7, Table 1), and the probability that a given taxa presents a given developmental mode (Figure 9, Table 3) are not influenced at all by the phylogenetic structure of our European species assemblage. This observation clearly points to a lack of any evolutionary constraint in the emergence of larval forms and their reversals to the non‐pelagic mode. In fact, the ability to delay larval hatching off the egg capsules until the time of metamorphosis facilitates the pelagic‐non‐pelagic transition (Page and Ferguson 2013; Collin et al. 2016). In essence, embryonic developmental time can be easily modulated to be completed inside or outside the capsule in response to environmental selective forces. Morphologically, the embryos inside the capsules of non‐pelagic developers are pelagic larvae, but they never get to the water column as they hatch after metamorphosis as crawling juveniles. Flexibility in the pelagic‐non‐pelagic transition is consistent with the observed poecilogony in some marine gastropods; that is, the coexistence of the 2 modes of development in the same species (Bouchet 1989; Przeslawski 2011; McDonald et al. 2014; Wiggering et al. 2020). The evolution of the egg capsule provides gastropods with a plasticity that is lacking in other marine invertebrates without capsules like most barnacles and bivalves. In these groups, the difficulties in removing larval forms once they are established in the life cycle may be reflected in the phylogeny, and in fact the phylogenetic footprint on developmental modes and other associated life history traits in barnacles is quite strong (Pechenik 1999; Ewers‐Saucedo and Pappalardo 2019). There are also constraints imposed by adult morphology, as some barnacles retain their naupliar larval stages within the mantle cavity, but at the high cost of delaying moulting (Anderson 1994). Similarly, most of the marine bivalves are broadcast spawners with external fertilisation, which prevents any trend towards larval retention inside adult morphological structures because there were never larvae to be retained in the first place (Young 2002). Conversely, marine gastropods circumvent these obstacles in the simplification of life cycles not by erasing larvae but just by retaining them throughout their whole development inside their egg capsules. Once pelagic larvae emerge in a given gastropod clade, the planktotrophic‐lecitotrophic transition is achieved by retaining larval feeding morphological adaptations even when they are not needed (Strathmann 1978). Such a strategy enables facultative planktotrophy in species that are usually lecithotrophic if a potential food source is available (Kempf and Hadfield 1985; Hadfield and Strathmann 1996). However, once achieved, lecithotrophic reversibility is not common as suggested by studies on lineage diversification in sea slugs and neogastropods (Krug et al. 2015; Friend et al. 2021).

Although the evolution of developmental mode is not related to phylogeny in gastropods, it clearly correlates with life‐history and morphological evolution. In general, our results suggest that non‐pelagic developers present larger initial embryo sizes but also larger juveniles (Figure 4). Consistently, the same pattern was found for Arctic bivalves (Thorson 1936, 1950) and, eventually, for the generality of marine invertebrates (Jaeckle 1995). To reach those large sizes, prolonged developmental times are required, too long for larval pelagic life to be selected, as they would entail massive mortality rates by predation and transport to unsuitable areas for settlement far from adult habitats (Byers and Pringle 2006; O'Connor et al. 2007; Pringle et al. 2014). On the other hand, smaller sizes of both early larvae and recently settled juveniles effectively shorten developmental time, making pelagic life possible. Such reductions in developmental time with smaller sizes arise from the non‐linear growth pattern in animals depicted by West 2001. Thus, for the same difference in initial and final sizes, developmental times are much shorter if both are small, as growth rates are much faster and the growth curve is much steeper at relatively small sizes (Bueno 2014; Vance 1973). This growth pattern could explain the absence of any effect of temperature consistent with organismal metabolism on the spatial distribution of developmental modes. On evolutionary time scales, cold temperatures may not pose a barrier to pelagic larval life, as a reduction in both initial and final larval sizes would suffice to reduce developmental times, thus compensating for the increase in larval duration driven by the slower organismal metabolism in colder waters (O'Connor et al. 2007). In agreement with these observations, the lecithotrophic species, which are the ones presenting significantly shorter developmental times (Figure 4G), are more prevalent in terms of species proportions in cold areas. This pattern is consistent with the negative effect of temperature in our SAR models (Figure 7B, Table 1) and with their mean proportions increasing northwards of the 40°–45° latitudinal band (Figure 6C), although both the proportions and the number of pixels available for their reliable calculation are too small to infer a real latitudinal trend. Nevertheless, poleward increments in the number of lecithotrophic molluscs and crustacean species have also been inferred from their latitudinal distribution ranges along the coasts of South America (Pappalardo and Fernández 2013). Moreover, a similar pattern was obtained globally for many different taxa (Marshall et al. 2012). Field samplings in Patagonia and Antarctica also point to a prevalence of the lecithotrophic mode in these cold environments (Pearse et al. 1991; Pearse 1994; Cañete et al. 2012). Lecithotrophic shorter pelagic larval durations are achieved not only by reducing initial and final larval sizes but also their ratio Gr1, which is significantly smaller with respect to planktotrophic species (Figure 4C). Such a reduction in pelagic duration could make maternal investment in nutritive reserves for larvae a cost‐effective strategy, thus leading to lecithotrophy. Consistently, facultative planktotrophy in lecithotrophic species can lead to an extended pelagic developmental time (Kempf and Hadfield 1985; Allen and Pernet 2007). In summary, the adaptation to metabolically driven thermal constraints in cold waters may not be the loss of the larval phase, but its shift to the lecithotrophic mode. More detailed and extensive spatial analyses are required in Europe, South America, and other poleward‐oriented continental margins to confirm these findings.

Nevertheless, when taken together, the life history traits showed a marked phylogenetic structure, as shown by the multivariate analyses (Table 2, Figure 8). Probably, these results are strongly influenced by food type, which is clearly associated with our gastropod phylogeny, at least at the superfamily level. Carnivorous species prevail within the large superfamilies Buccinoidea, Muricoidea and Naticoidea, while those within Patelloidea, Littorinoidea and Trochoidea are almost exclusively herbivorous. Such dietary‐phylogenetic coupling may reflect evolutionary morphological constraints in the adaptation of the radula and digestive apparatus to new food sources (Declerck 1995; Page 2000; Vortsepneva et al. 2022).

### 4.3 Environmental Drivers Behind the Pelagic–Non‐Pelagic Transition

Our results suggest that the metabolically driven effects of temperature on developmental time are not responsible for the evolutionary loss of pelagic larvae. On the contrary, we found that other 2 very different environmental variables, not strictly determined by latitude, may drive such reversible transition: habitat depth and the seasonality of primary productivity. Depth clearly affects the pattern of interspecific variability in morphological and life‐history traits (Figure 8, Table 2), while both the spatially‐explicit proportions and the occurrence probability of non‐pelagic developers decreases in shallow environments (Figures 7 and 9, Tables 1 and 3). More specifically, SAR models suggest that pelagic development can take place at all depths, but non‐pelagic development is restricted to deeper habitats (Figure 7, Table 1). Therefore there are strong selective forces correlating with habitat depth driving the elimination of non‐pelagic development in shallow water. From the intertidal to the outer shelf, planktotrophy has been shown to decrease in molluscs (Jackson 1974; Bandel 1975; Jablonski and Lutz 1983). Thorson also studied the apparent increase in non‐pelagic developers in deep seas and concluded that limited food and cold temperatures were behind this pattern (Thorson 1950). However, we think that the most obvious correlate of depth that could be behind this pattern is habitat temporal stability. There is a large body of research on larval behaviours, distributions, genetic patterns and biogeography that clearly shows how dispersal by juveniles, egg masses and other benthic stages of non‐pelagic developers generally overcomes pelagic larval dispersal in all these aspects (Slatkin 1985; Johannesson 1988; Martel and Chia 1991; Pineda et al. 2007; Cowen and Sponaugle 2009). However, careful reviews of larval biology across many different taxonomic groups together with recent mathematical models show that if benthic populations are established in non‐permanent habitats frequently disturbed or even destroyed, then planktonic larvae become essential for the survival of a metapopulation (Mileikovsky 1971; Barlow 1981; Iwasa et al. 2022). In essence, the intrinsic advantage of the non‐pelagic mode in keeping individuals within a given delimited population turns into a disadvantage if this population disappears. Habitat turnover rates depend on the variability of sedimentary processes that usually slow down with depth as bottom currents decrease and the coastal source of sedimentary materials gets farther away (Milliman and Syvitski 1992). Recent predictive machine learning models have calculated marine sedimentation patterns on a global scale that are consistent with a bathymetric negative effect on vertical sedimentation rates (Restreppo et al. 2020). In addition, studies on coral reef dynamics have concluded that the disturbances produced by storms and drastic shifts in salinity and temperature are all attenuated by water column depth (Liddell and Ohlhorst 1988; Glynn 1996; Bongaerts et al. 2010). These characteristics increase habitat stability in deep benthic environments and are thought to be behind the reduction in population differentiation, speciation rates and species richness in these regions (Etter and Rex 1990; Costello and Chaudhary 2017). Therefore, the retention of individuals within their own local population provided by a non‐pelagic development may be selected at these stable habitats. Conversely, highly dynamic bottom habitats with increased turnover rates in shallow environments may be an important selective force favouring the appearance of larval forms in the life cycles of many marine invertebrates. Consistently, a high proportion of benthic invertebrates present dispersive larvae in Antarctic shallow environments subjected to frequent bottom disturbances by anchor ice formation (Pearse et al. 1991). Still, larval behaviours in the water column of most species tend to enhance onshore retention, thus being suitable for alongshore, relatively short scale dispersal in between short lived patches of good quality benthic habitat (Sponaugle et al. 2002; Cowen et al. 2006; Becker and Levin 2007; Burgess et al. 2015). Nevertheless, in prosobranchs planktotrophy was found to increase beyond the shelfbreak to the abyssal plains in the western North Atlantic, a pattern that may be explained by stronger selection for dispersive larvae connecting very distant and isolated populations (Rex and Waren 1982). The difference in depth ranges between that study (478–4970 m) and ours (0–1022 m) prevents for any realistic comparison.

As a by‐product of the association between depth and developmental mode, we found that carnivorous feeding was correlated with non‐pelagic developers (Figure 8A). At deep stable habitats where larval forms are not produced, the lack of macroalgae and other primary producers also drives the evolution of carnivores. Accordingly, a decline of herbivore gastropod species with depth was observed at all latitudes along the northeastern Pacific (Valentine et al. 2002).

Seasonality of primary productivity has also been identified as a potential driver of the pelagic‐non‐pelagic transition according to its clear effect on the association between the different life history traits (Figure 8, Table 2), developmental mode probability (Figure 9 Table 3), and to a lesser extent on the spatial distribution of developmental mode proportions (Figure 7, Table 1). The results of these analyses all point to a consistent pattern: a prevalence of the pelagic larval mode with a decreased seasonality of primary productivity inferred as the Colwell's contingency index calculated on chlorophyll‐a time series. Interestingly, our SAR models revealed that lower M_CHLA estimates correlated with the spatially explicit probabilities of feeding planktotrophic species, but this effect was not observed in lecithotrophic taxa (Figure 7, Table 1). In agreement with these patterns, analyses of the fossil record in marine echinoids show that the simultaneous emergence of non‐planktotrophic developmental modes in different phylogenetic groups coincided with the dawn of seasonal primary productivity in the Cretaceous period (Jeffery 1997; Jeffery et al. 2003). Moreover, to some extent Thorson attributed the apparent dominance of non‐pelagic developers in Nordic seas to a brief seasonal period of high primary productivity (Thorson 1950). Thus, the probability of a temporal mismatch between the timing of the chlorophyll‐a seasonal peak and that of spawning in planktotrophic species increases with a shortening of the period of high phytoplankton abundance (Wray 1996). In this scenario, non‐planktotrophic modes would evolve as a response to the negative selective force this mismatch exerts on planktotrophic species.

Although we believe that Thorson's hypothesis regarding the effects of primary productivity on larval development is essentially correct, our results suggest that highly seasonal phytoplankton life cycles are not exclusive of Nordic seas and, therefore, are not behind any latitudinal trend. In fact, the spatial distribution of M_CHLA indicates that chlorophyll‐a seasonality actually peaks within the Mediterranean basin and in the subtropical Atlantic (Figure 2D). Remarkably, our M_CHLA distribution matches the spatial pattern of seasonal variance in phytoplankton phenology in the Mediterranean Sea obtained from satellite derived chl‐a time series from 1998 to 2014, thus partially overlapping with our 2003–2022 time series (Salgado‐Hernanz et al. 2019). Both studies, using different methodologies, consistently show an enhanced seasonality in the Eastern Mediterranean compared to the western basin, even capturing sub‐mesoscale features like a 200 km wide gap of reduced seasonal fluctuations to the southwest of Turkey (Figure 2D in the current manuscript and Figure 3A in Salgado‐Hernanz et al. 2019). Essentially, these spatial distributions reflect the strong chlorophyll‐a seasonality within the oligotrophic subtropical latitudinal band in between 30° and 40° N, characterised by a sharp autumn/winter phytoplankton bloom (González Taboada and Anadón 2014). In fact, the probability of bloom occurrence in spring off Iceland is lower than the probability of bloom occurrence in wintertime in the oligotrophic region, and further north towards Greenland there is not even a clear identifiable seasonal pattern (González Taboada and Anadón 2014), thus matching our M_CHLA spatial patterns. On a global scale, Marshall and Burguess (2014) failed to find any simple latitudinal pattern in the distribution of primary productivity seasonality also calculated as Colwell's M index. However, their results point to a prevalence of species with planktonic larvae in chlorophyll‐a seasonal waters, although the evolutionary drivers of such relationship remain unclear.

The only primary productivity variable that shows a clear latitudinal trend is CHLA_MAX, which increases steadily northwards along the European Atlantic facade towards the Baltic Sea (Figure 2B). Seasonality of food availability was found to be more important than its magnitude in determining the spatiotemporal distributions of marine benthic invertebrates (Starr et al. 1990; Muñiz et al. 2021). Furthermore, contrary to Thorson's rule, our gastropod dataset indicates that there are several non‐pelagic developers inhabiting relatively highly seasonal Mediterranean waters with M_CHLA > 0.2 like the cassid *Galeodea echinophora*, the muricids *Bolinus brandaris* and *Hexaplex trunculus*, the conid *Conus ventricosus*, the cerithid *Thericium repandum* and the fasciolarid *Pseudofusus rostratus*, among others. Given the poleward expansion of the oligotrophic seasonal band with a wintertime bloom (González Taboada and Anadón 2014), a northward distribution shift of these species is plausible.

Interestingly, water column circulation variables significantly affected the spatial distribution of the developmental mode proportion of pelagic planktotrophic gastropods (Figure 7, Table 1). Noteworthy, this effect was detected only in those species whose larvae spend more time facing the currents in the pelagic environment, as lecithotrophic species typically presented short‐lived larvae (Figure 4). Specifically, planktotrophic proportions increase with weaker – although strongly stratified – currents, a pattern entirely consistent with well‐established knowledge on larval ecology and distributions. Highly vertically flow‐stratified water columns in two or more layers moving in opposing directions make larval vertical migration effective to avoid offshore transport for different taxa across a wide range of systems and spatiotemporal scales, including the coasts of the Red Sea (Genin et al. 2005), internal wave and topographic fronts worldwide (Pineda 1999; Shanks et al. 2003; Weidberg et al. 2014, 2019), meso‐scale upwelling–downwelling transitions at the main upwelling systems of the world (Shanks and Shearman 2009; Miller and Morgan 2013) and estuarine systems (Queiroga and Blanton 2004). Without such stratification, larvae are forced to passively drift with the currents, no matter their vertical active positioning, and this is translated to massive offshore advection by uniform, strong flows like the Gulf Stream and the Agulhas Current (Graber and Limouzy‐Paris 1997; Weidberg et al. 2015) and eventually to recruitment failures at coastal habitats driven by vertically uniform warm anomalies like The Blob and El Niño (Pineda and López 2002; Hagerty et al. 2018). Water column uniform warmings could result both in homogeneous flows and the loss of thermal stratification (Hagerty et al. 2018), but on the other hand, circulation in two layers has also been observed even in mixed shallow waters with no thermocline (Kirincich et al. 2005). Therefore, it may not be possible to derive global or even regional patterns in dynamic stratification like the ones used here (Figure 1D) from thermal stratification and/or surface temperatures, and in turn, the use of complex but reliable hydrographical models based on empirical observations is required. Overall, our results lend support to the idea that planktonic larvae are favoured by circulation features which in conjunction with their own behaviour allow their onshore retention and a reduction in their dispersal distances. Consistently, Pringle et al. (2014) found that lower proportions of planktotrophers occurred as mean currents increased for many different taxa on a global scale. We have now expanded this line of evidence by including for the first time the dynamic stratification of the water column in this kind of biogeographical studies and assessing its significant effects.

## 5 Conclusions

Thanks to the very detailed spatial analyses performed on the distributions of a wide range of European gastropods, we confirm that Thorson's latitudinal trend towards the prevalence of non‐pelagic developers (historically termed as Thorson's rule) is an artefact derived from a geographically restricted sampling. After accounting for a larger number of species and sampling coverage to analyse spatially explicit developmental mode proportions, Thorson's rule disappears for marine gastropods. We also explored the environmental drivers behind the probability of presenting a given developmental mode. We have found that habitat depth and primary productivity play a fundamental role, and they do not co‐vary with latitude. Planktonic larvae may be favoured in shallow, temporally unstable habitats where a short‐range dispersal is selected to overcome frequent local extinctions. On the other hand, seasonal phytoplankton blooms increase the probability of mismatch in the occurrence of larvae and their food in the pelagic environment, thus making non‐pelagic development a safer strategy. Furthermore, we observed that spatially explicit proportions of planktotrophic developers increase with weaker and more vertically stratified currents, thus showing that the evolution of pelagic larval forms is favoured when circulation features enable nearshore retention. Developmental modes are not influenced by gastropod phylogeny, probably because the evolution of egg capsules makes the pelagic‐non‐pelagic transition reversible regardless of the gastropod lineage. In contrast, developmental modes correlate with other life history and morphological traits, especially with developmental times, larval initial and final sizes and their ratio, all of them significantly lower in lecithotrophic developers, probably as an adaptation to cold environments. All in all, if a latitudinal trend driven by thermal effects on pelagic larval duration exists, it is towards more frequent lecithotrophic species in Nordic seas, not towards more non‐pelagic developers.

## Author Contributions

**Nicolás Weidberg:** conceptualization (lead), data curation (lead), formal analysis (lead), investigation (lead), methodology (lead), supervision (lead), validation (lead), visualization (lead), writing – original draft (lead), writing – review and editing (supporting). **Juan Bueno‐Pardo:** conceptualization (equal), formal analysis (supporting), methodology (equal), supervision (lead), writing – original draft (supporting), writing – review and editing (lead). **Ainhoa de Diego:** conceptualization (supporting), data curation (equal), formal analysis (supporting), investigation (supporting), supervision (supporting), validation (lead), visualization (supporting), writing – review and editing (supporting). **José Luis Acuña:** conceptualization (supporting), formal analysis (supporting), investigation (equal), methodology (equal), supervision (lead), visualization (supporting), writing – original draft (supporting), writing – review and editing (lead).

## Conflicts of Interest

The authors declare no conflicts of interest.

## Supporting information


**Appendix S1:** ece373147‐sup‐0001‐AppendixS1.pptx.

**Appendix S2:** ece373147‐sup‐0002‐AppendixS2.docx.

## Data Availability

In accordance with the recommendations to support open access to scientific information international, the European Community and national level, the University of Oviedo and the University of Vigo will promote the open access deposit of the research work of its teaching and research staff. Data are provided as private‐for‐peer review (click here to visualise DATA). The University of Oviedo online and GitHub repositories will be used for data storage and open access. No novel code is provided in this paper. https://raw.githack.com/NicoW82/MI_HTML/main/DATA%20OK%20worms%20FINAL%202.htm.
