# Supplementary material for: Primary Productivity and Habitat Depth Shape Developmental Mode in European Marine Gastropods
Source: Ecol Evol. 2026 Mar 8;16(3):e73147. doi: 10.1002/ece3.73147 (PMC12967624; doi:10.1002/ece3.73147)
Supplement: Supplementary file 1 — Appendix S1: ece373147‐sup‐0001‐AppendixS1.pptx. [file ECE3-16-e73147-s001.pptx]

## Slide 1
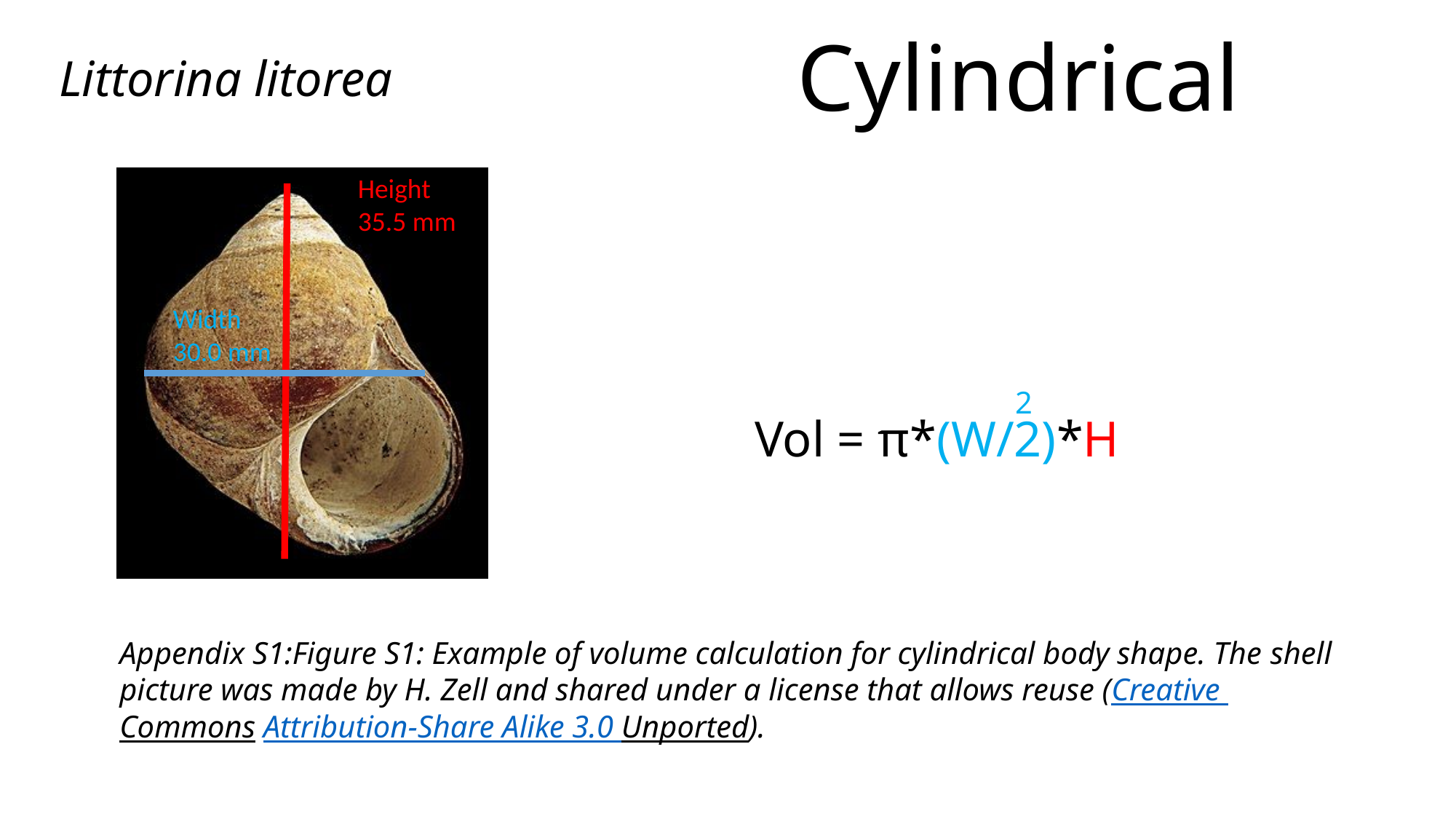

Littorina litorea
Cylindrical
Height
35.5 mm
Width
30.0 mm
2
Vol = π*(W/2)*H
Appendix S1:Figure S1: Example of volume calculation for cylindrical body shape. The shell picture was made by H. Zell and shared under a license that allows reuse (Creative Commons Attribution-Share Alike 3.0 Unported).

## Slide 2
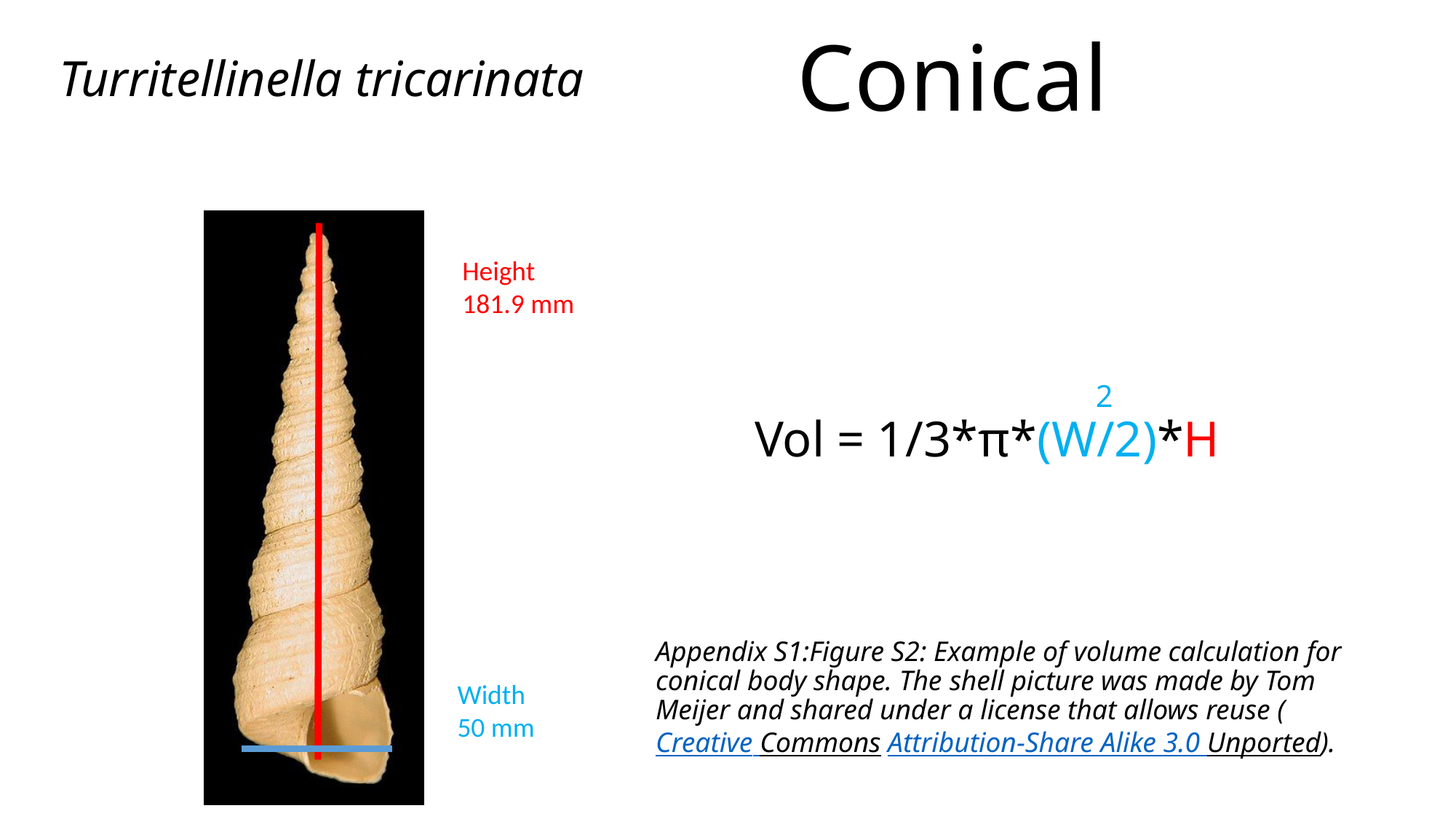

Turritellinella tricarinata
Conical
Height
181.9 mm
2
Vol = 1/3*π*(W/2)*H
Appendix S1:Figure S2: Example of volume calculation for conical body shape. The shell picture was made by Tom Meijer and shared under a license that allows reuse (Creative Commons Attribution-Share Alike 3.0 Unported).
Width
50 mm

## Slide 3
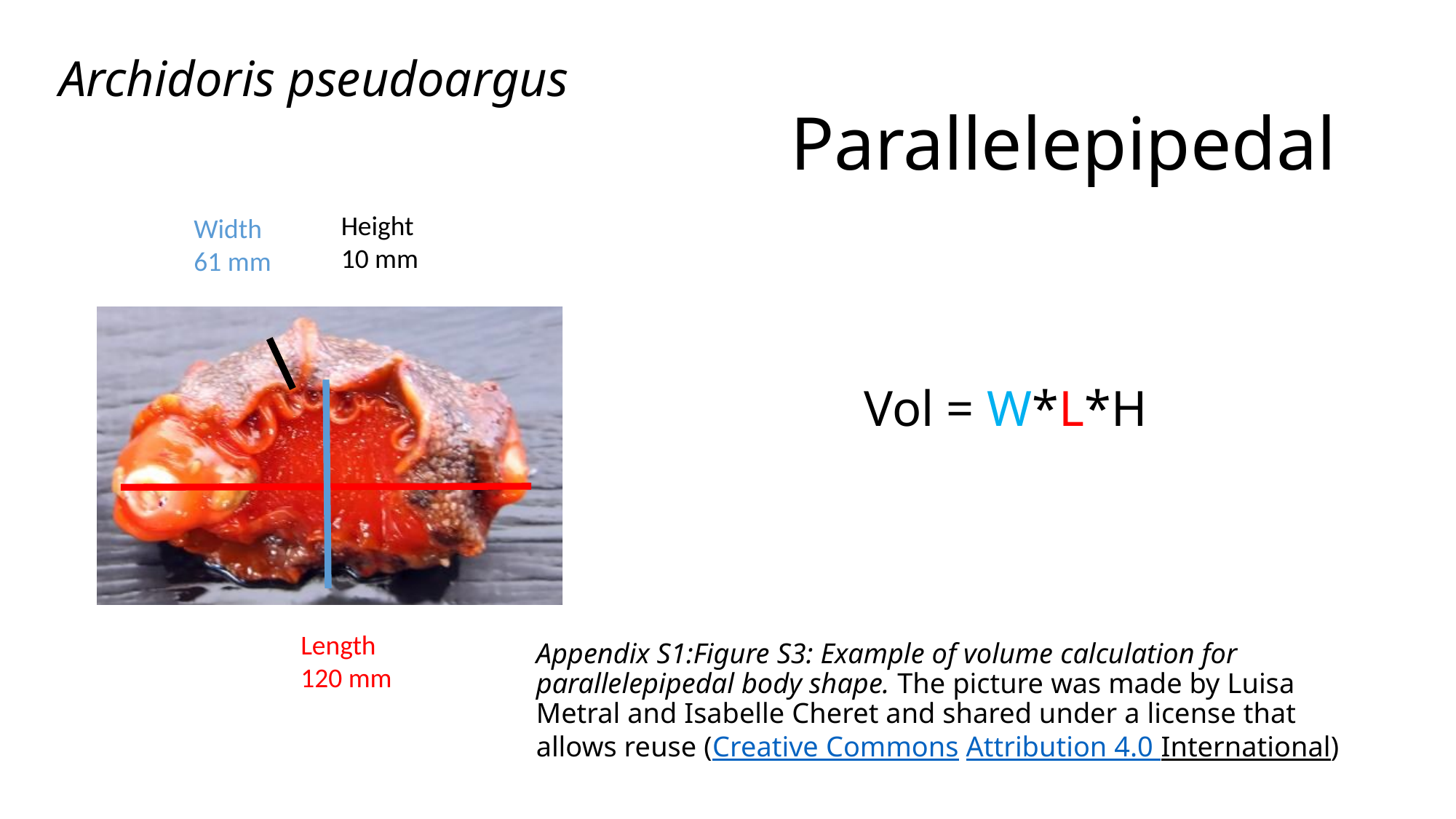

Archidoris pseudoargus
Parallelepipedal
Height
10 mm
Width
61 mm
Vol = W*L*H
Length
120 mm
Appendix S1:Figure S3: Example of volume calculation for parallelepipedal body shape. The picture was made by Luisa Metral and Isabelle Cheret and shared under a license that allows reuse (Creative Commons Attribution 4.0 International)

## Slide 4
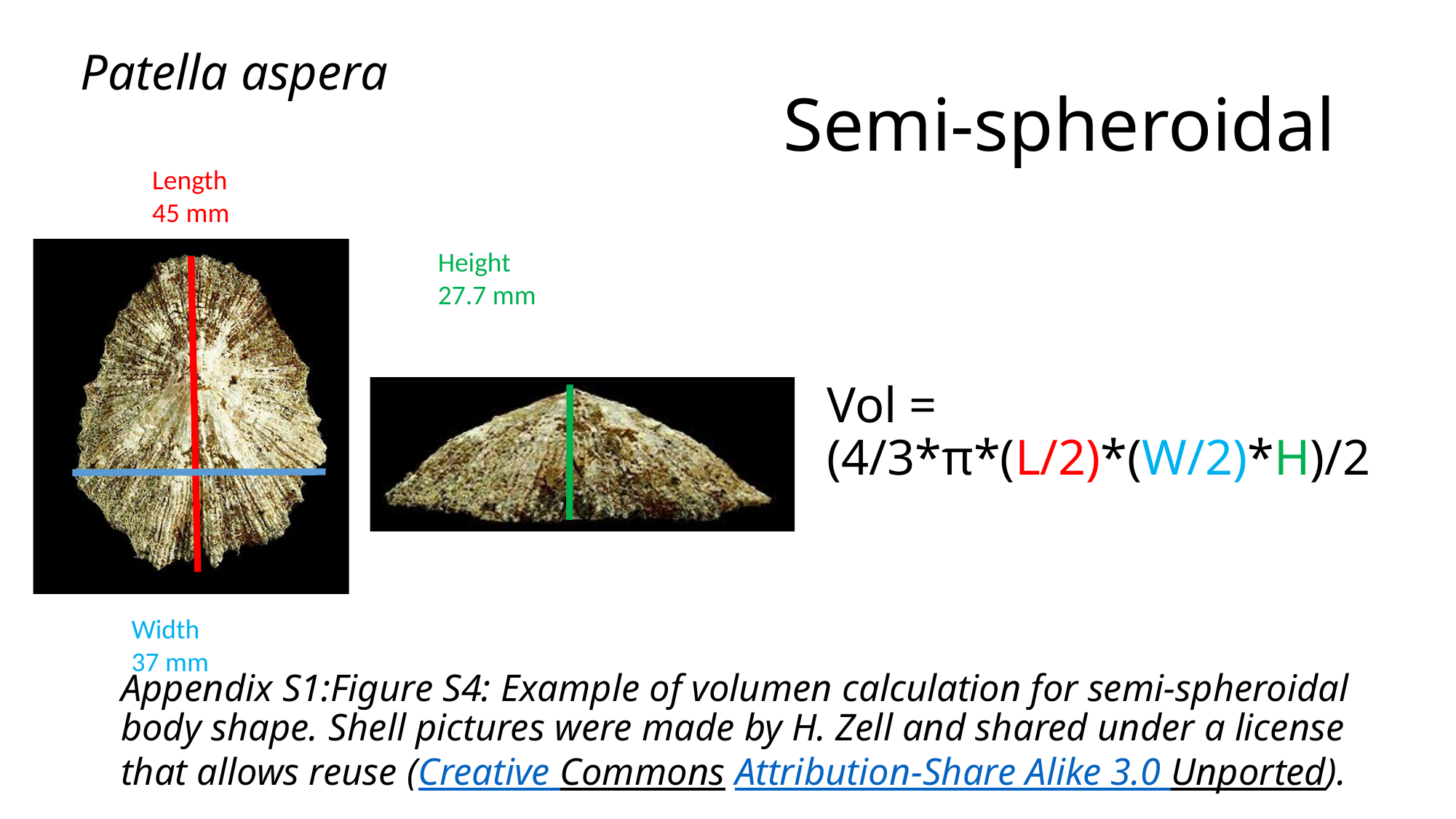

Patella aspera
Semi-spheroidal
Length
45 mm
Height
27.7 mm
Vol = (4/3*π*(L/2)*(W/2)*H)/2
Width
37 mm
Appendix S1:Figure S4: Example of volumen calculation for semi-spheroidal body shape. Shell pictures were made by H. Zell and shared under a license that allows reuse (Creative Commons Attribution-Share Alike 3.0 Unported).
