# Supplementary material for: Primary Productivity and Habitat Depth Shape Developmental Mode in European Marine Gastropods
Source: Ecol Evol. 2026 Mar 8;16(3):e73147. doi: 10.1002/ece3.73147 (PMC12967624; doi:10.1002/ece3.73147)
Supplement: Supplementary file 2 — Appendix S2: ece373147‐sup‐0002‐AppendixS2.docx. [file ECE3-16-e73147-s002.docx]

Appendix S2

The relationship between developmental times and the temperature-size metabolic term is presented in Figure1. This analysis was based on the database shown in Table 1. The linear fit without the intercept term allows for the calculation of the (4/a_0_) constant in Equation 1 in the main manuscript. This constant presents a value of 5.796EXP-10 days/mm^0.75^.





Appendix S2: Figure S1. Linear regression between developmental time and the temperature-size term

| Species | DT, days | Temperature, Cº | IS, um | JS, um | ΔS, mm^3^ | Temperature-size metabolic term, mm ^0.75*°K^ | References |
| --- | --- | --- | --- | --- | --- | --- | --- |
| *Crepidula plana* | 71.9 | 12 | 834.6 | 843.6 | 0.00995394 | 96411586715.252 | Lima and Pechenik 1985 |
| *Crepidula plana* | 24.8 | 20 | 751.98 | 1043.9 | 0.37298183 | 115914914464.511 | Lima and Pechenik 1985 |
| *Crepidula plana* | 20.6 | 25 | 728.7 | 1077 | 0.45150088 | 78984125981.149 | Lima and Pechenik 1985 |
| *Crepidula plana* | 14.47 | 29 | 738.72 | 1040 | 0.37781767 | 54048813804.638 | Lima and Pechenik 1985 |
| *Crepidula plana* | 22.7 | 25 | 54.7 | 127.2 | 0.00099191 | 17099896215.099 | Pechenik et al., 1996 |
| *Crepidula plana* | 19 | 25 | 50.6 | 154 | 0.00184449 | 19968447119.778 | Pechenik et al., 1996 |
| *Crepidula plana* | 9.6 | 25 | 56.4 | 139.9 | 0.00133974 | 18434471213.993 | Pechenik et al., 1996 |
| *Crepidula plana* | 8.6 | 25 | 68.9 | 156.9 | 0.00185115 | 19986435074.273 | Pechenik et al., 1996 |
| *Crepidula fornicata* | 30 | 25 | 70.8 | 119.5 | 0.0007077 | 15715808104.622 | Pechenik et al., 1996 |
| *Littorina littorea* | 28 | 25 | 80 | 250 | 0.00791317 | 28738410682.760 | Struhsaker and Costlow 1968 |
| *Nassarius obsoletus* | 12 | 25.1 | 280 | 657 | 0.13699543 | 58125796183.989 | Scheltema 1967 |
| *Nassarius obsoletus* | 12 | 17.3 | 280 | 436 | 0.03190287 | 79619893528.583 | Scheltema 1967 |
| *Nassarius obsoletus* | 12 | 25 | 280 | 655 | 0.13564349 | 58475629329.046 | Scheltema 1967 |
| *Nassarius obsoletus* | 12 | 17.2 | 280 | 498 | 0.05317367 | 91279146579.875 | Scheltema 1967 |
| *Nassarius obsoletus* | 13 | 24.5 | 280 | 698 | 0.16656574 | 64227730965.305 | Scheltema 1967 |
| *Nassarius obsoletus* | 13 | 17.7 | 280 | 488 | 0.04935573 | 85682507463.937 | Scheltema 1967 |
| *Nassarius obsoletus* | 12 | 26.4 | 268 | 672 | 0.1488153 | 53174323507.682 | Scheltema 1967 |
| *Nassarius obsoletus* | 12 | 17.5 | 268 | 496 | 0.05381304 | 89130741443.013 | Scheltema 1967 |
| *Nassarius obsoletus* | 12 | 26.4 | 268 | 672 | 0.1488153 | 53174323507.682 | Scheltema 1967 |
| *Nassarius obsoletus* | 12 | 17.3 | 268 | 492 | 0.05227971 | 90083953065.505 | Scheltema 1967 |
| *Nassarius obsoletus* | 17 | 23.9 | 271 | 615 | 0.11137279 | 61128552713.219 | Scheltema 1967 |
| *Nassarius obsoletus* | 17 | 15.8 | 271 | 447 | 0.03634418 | 94124805105.821 | Scheltema 1967 |
| *Nassarius obsoletus* | 12 | 23.8 | 262 | 529 | 0.06809479 | 54518060927.585 | Scheltema 1967 |
| *Nassarius obsoletus* | 12 | 15.9 | 262 | 422 | 0.02993249 | 88860778356.328 | Scheltema 1967 |
| *Nassarius obsoletus* | 9 | 24.6 | 268 | 463 | 0.0418901 | 45098163568.876 | Scheltema 1967 |
| *Nassarius obsoletus* | 9 | 16.5 | 268 | 361 | 0.01455453 | 70299937217.334 | Scheltema 1967 |
| *Nassarius obsoletus* | 12 | 25.3 | 268 | 589 | 0.09691189 | 52411731671.403 | Scheltema 1967 |
| *Nassarius obsoletus* | 12 | 16.6 | 268 | 413 | 0.02680631 | 81163836284.909 | Scheltema 1967 |
| *Concholepas concholepas* | 92.5 | 20 | 250 | 1750 | 2.7979875 | 191835440176.261 | Campos et al., 1994 |
| *Conus texile* | 15 | 25 | 530 | 1510 | 1.72477715 | 110422713182.791 | Perron 1981 |
| *Conus texile* | 16 | 25 | 530 | 1510 | 1.72477715 | 110422713182.791 | Perron 1981 |
| *Conus lividus* | 50 | 25 | 250 | 1380 | 1.36787725 | 104204613772.590 | Perron 1981 |
| *Conus abbreviatus* | 32 | 25 | 270 | 1120 | 0.72531428 | 88921488499.066 | Perron 1981 |
| *Conus flavius* | 23 | 25 | 317 | 1230 | 0.95767068 | 95318942188.916 | Perron 1981 |
| *Conus quercinus* | 30 | 25 | 282 | 1325 | 1.20625782 | 100979961898.702 | Perron 1981 |
| *Conus striatus* | 20 | 25 | 488 | 1552 | 1.89652859 | 113074588365.038 | Perron 1981 |
| *Conus marmoreus* | 10 | 25 | 755 | 1482 | 1.47895181 | 106258497979.510 | Perron 1981 |
| *Charonia tritonis* | 59 | 24 | 428 | 700 | 0.13854312 | 64008180604.737 | Zhang et al., 2013 |
| *Lithopoma undosa* | 30 | 20 | 125 | 600 | 0.11207494 | 85821190129.600 | Salas-Garza et al., 2009 |
| *Crepidula fornicata* | 12 | 25 | 295 | 365 | 0.01201911 | 31903859050.134 | Lucas and Costlow 1979 |
| *Tegula rustica* | 7 | 20 | 140 | 230 | 0.00493388 | 39311046713.776 | Kulikova and Omelyamenko 2000 |
| *Aplysia dactylomela* | 30 | 26 | 144 | 313 | 0.01449236 | 30721543503.690 | Switzer-Dunlap and Hadfield 1977 |
| *Aplysia juliana* | 28 | 26 | 125 | 322 | 0.01645838 | 31714291272.392 | Switzer-Dunlap and Hadfield 1977 |
| *Dolabella ouricularia* | 31 | 26 | 148 | 295 | 0.01174465 | 29148638083.585 | Switzer-Dunlap and Hadfield 1977 |
| *Stylocheilus longicauda* | 30 | 26 | 103 | 333 | 0.01876232 | 32770257898.009 | Switzer-Dunlap and Hadfield 1977 |
| *Tegula funebralis* | 40 | 20 | 223 | 463 | 0.04616229 | 68752618726.231 | Guzman Del Proo et al., 2007 |
| *Dicathais orbita* | 23 | 16 | 310.01 | 317.03 | 0.00108394 | 38415613957.507 | Noble et al., 2015 |
| *Dicathais orbita* | 23 | 22 | 575.38 | 866.48 | 0.24088527 | 87291384855.020 | Noble et al., 2015 |
| *Charonia sguenzae* | 74 | 17 | 361 | 602 | 0.08959913 | 105876095976.163 | Doxa et al., 2021 |
| *Charonia sguenzae* | 35 | 20 | 363 | 663 | 0.12755006 | 88641595982.812 | Doxa et al., 2021 |
| *Charonia sguenzae* | 49 | 23 | 354 | 666 | 0.13144791 | 68824231724.768 | Doxa et al., 2021 |
| *Thais haemastoma canaliculata* | 27 | 24 | 147.9 | 872.85 | 0.34649779 | 80494157858.328 | Dobberteen and Pechenik 1987 |
| *Crepidula fornicata* | 6 | 24 | 384.91 | 776.83 | 0.2155991 | 71490935149.439 | Dobberteen and Pechenik 1987 |
| *Strombus costatus* | 35 | 24 | 323.48 | 709.18 | 0.16903045 | 67271401923.043 | Aldana Aranda et al., 1989 |
| *Strombus costatus* | 26 | 28 | 297.16 | 715.5 | 0.17805165 | 48651065712.183 | Aldana Aranda et al., 1989 |
| *Strombus costatus* | 28 | 28 | 297.52 | 685.88 | 0.15515501 | 47005351810.660 | Aldana Aranda et al., 1989 |
| *Strombus gigas* | 29 | 29 | 290 | 883 | 0.3477104 | 52938303400.105 | Brito-Manzano and Aldana Aranda 2004 |
| *Strombus gigas* | 29 | 29 | 300 | 867 | 0.32710044 | 52135780783.882 | Brito-Manzano and Aldana Aranda 2004 |
| *Strombus gigas* | 29 | 29 | 300 | 881 | 0.34389935 | 52792647047.549 | Brito-Manzano and Aldana Aranda 2004 |
| *Strombus gigas* | 27 | 29 | 300 | 882 | 0.34511993 | 52839428149.346 | Brito-Manzano and Aldana Aranda 2004 |
| *Strombus gigas* | 25 | 29 | 300 | 894 | 0.35998389 | 53399398727.314 | Brito-Manzano and Aldana Aranda 2004 |
| *Strombus gigas* | 27 | 29 | 300 | 904 | 0.37267925 | 53864099217.478 | Brito-Manzano and Aldana Aranda 2004 |
| *Strombus gigas* | 29 | 29 | 300 | 902 | 0.37011756 | 53771298083.452 | Brito-Manzano and Aldana Aranda 2004 |
| *Strombus canarium* | 25 | 29 | 224.83 | 950.19 | 0.44324033 | 56250374479.036 | Cob et al., 2009 |
| *Strombus canarium* | 25 | 29 | 210.38 | 1343.1 | 1.26358283 | 73091441780.937 | Cob et al., 2009 |
| *Rapana venosa* | 20 | 25 | 344 | 768 | 0.21586837 | 65678377905.572 | Ban et al., 2014 |
| *Rapana venosa* | 20 | 25 | 380 | 886 | 0.3354362 | 73329318601.411 | Ban et al., 2014 |
| *Rapana venosa* | 20 | 25 | 372 | 1043 | 0.56713611 | 83617464118.171 | Ban et al., 2014 |
| *Rapana venosa* | 20 | 25 | 384 | 1193 | 0.85939146 | 92773282991.778 | Ban et al., 2014 |
| *Rapana venosa* | 15 | 25 | 388 | 870 | 0.31420813 | 72140562179.718 | Ban et al., 2014 |
| *Rapana venosa* | 15 | 25 | 362 | 863 | 0.31169789 | 71996043510.134 | Ban et al., 2014 |
| *Rapana venosa* | 15 | 25 | 338 | 789 | 0.23695759 | 67226859135.723 | Ban et al., 2014 |
| *Strombus pugilis* | 31 | 29 | 212 | 997 | 0.5139128 | 58369766685.445 | Brito-Manzano et al., 1999 |
| *Tegula eiseni* | 63 | 20 | 160 | 610 | 0.11670259 | 86693696358.321 | Guzman Del Proo et al., 2011 |
| *Crepipatella peruviana* | 15 | 17 | 422.5 | 650 | 0.10430432 | 109975905753.766 | Chaparro et al., 2005 |
| *Fusitriton magellanicus* | 63.85 | 10 | 180 | 296 | 0.01052558 | 117845360544.309 | Cañete et al., 2012 |
| *Charonia tritonis* | 300 | 27.75 | 822.5 | 2000 | 3.89745514 | 107444967474.351 | Nugranad et al., 2021 |
| *Cymatium nicobaricum* | 320 | 22.5 | 1200 | 4600 | 50.0603488 | 317415166169.357 | Scheltema 1971 |
| *Cymatium parthenopeum* | 293 | 22.5 | 600 | 3000 | 14.0241024 | 230926075170.796 | Scheltema 1971 |

**Appendix S2: Table S1. Developmental times, initial larvae and juveniles sizes, and rearing temperatures for a collection of gastropod species worldwide**

**References**

**Aldana Aranda, D., L. Lucas, T. Brulé, E. Salguero, and F. Rendón.** 1989. Effects of temperature, algal food, feeding rate and density on the larval growth of the milk conch (Strombus costatus) in Mexico. Aquaculture 76:361–371.

**Ban, S., T. Zhang, H. Pan, Y. Pan, P. Wang, and D. Xue.** 2014. Effects of temperature and salinity on the development of embryos and larvae of the veined rapa whelk Rapana venosa (Valenciennes, 1846). Chinese Journal of Oceanology and Limnology 32:773–782.

**Campos, E. O., A. Pinto, E. Bustos, S. R. Rodriguez, and N. C. Inestrosa.** 1994. Metamorphosis of laboratory-reared larvae of Concholepas concholepas (Mollusca; Gastropoda). Aquaculture 126:299–303.

**Cañete, J. I., C. S. Gallardo, T. Céspedes, C. A. Cárdenas, and M. Santana.** 2012. Encapsulated development, spawning and early veliger of the ranellid snail Fusitriton magellanicus (Röding, 1798) in the cold waters of the Magellan Strait, Chile. Latin American Journal of Aquatic Research 40:914–928. https://doi.org/10.3856/vol40-issue4-fulltext-8

**Chaparro, O. R., C. L. Saldivia, S. V. Pereda, C. J. Segura, Y. A. Montiel, and R. Collin.** 2005. The reproductive cycle and development of Crepipatella fecunda (Gastropoda: Calyptraeidae) from southern Chile. Journal of the Marine Biological Association of the United Kingdom 85:157–161. <https://doi.org/10.1017/S0025315405010982h>

**Doxa, C. K., A. Sterioti, P. Divanach, and M. Kentouri.** 2021. Effect of temperature on embryonic development of the marine gastropod Charonia seguenzae (Aradas & Benoit, 1870). Journal of Thermal Biology 100:103044.

**Dobberteen, R. A., and J. A. Pechenik.** 1987. Comparison of larval bioenergetics of two marine gastropods with widely differing lengths of planktonic life, Thais haemastoma canaliculata (Gray) and Crepidula fornicata (L.). Journal of Experimental Marine Biology and Ecology 109:173–191.

**Gail, M. L., and J. A. Pechenik.** 1985. The influence of temperature on growth rate and length of larval life of the gastropod, Crepidula plana Say. Journal of Experimental Marine Biology and Ecology 90:55–71.

**Guzmán del Próo, S. A., T. Reynoso-Granados, E. Serviere-Zaragoza, and P. Monsalvo-Spencer.** 2011. Larval and early juvenile development of Tegula eiseni (Jordan, 1936) (Gastropoda: Trochidae). CICIMAR Oceánides 26:43–50.

**Guzmán del Próo, S. A., T. Reynoso-Granados, P. Monsalvo-Spencer, and E. Serviere-Zaragoza.** 2006. Larval and early juvenile development in Tegula funebralis (Adams, 1855) (Gastropoda: Trochidae) in Baja California Sur, México. The Veliger 48:116–120.

**Kulikova, V. A., and V. A. Omel'yanenko.** 2000. Reproduction and larval development of the gastropod mollusk Tegula rustica in Peter the Great Bay, Sea of Japan. Russian Journal of Marine Biology 26:128–130.

**Lucas, J. S., and J. D. Costlow.** 1979. Effects of various temperature cycles on the larval development of the gastropod mollusc Crepidula fornicata. Marine Biology 51:111–117. <https://doi.org/10.1007/BF00555190>

**Noble, W. J., K. Benkendorff, and J. O. Harris.** 2015. Growth, settlement and survival of Dicathais orbita (Neogastropoda, Mollusca) larvae in response to temperature, diet and settlement cues. Aquaculture Research 46:1455–1468.

**Nugranad, I., K. Promjinda, T. Varapibal, and S. Chantara.** 2001. Reproduction of the trumpet triton Charonia tritonis (Mollusca: Gastropoda) in captivity. Phuket Marine Biological Center Special Publication 25:153–160.

**Perron, F. E.** 1981. Larval biology of six species of the genus Conus (Gastropoda: Toxoglossa) in Hawaii, USA. Marine Biology 61:215–220. <https://doi.org/10.1007/BF00386662>

**Pechenik, J. A., T. J. Hilbish, L. S. Eyster, and D. Marshall.** 1996. Relationship between larval and juvenile growth rates in two marine gastropods, Crepidula plana and C. fornicata. Marine Biology 125:119–127.

**Salas-Garza, A., G. Parés-Sierra, R. Gómez-Rigalt, and E. Carpizo-Ituarte.** 2009. The larval development, metamorphosis and juvenile growth of the turban snail Lithopoma (Astraea) undosa (Wood, 1828) (Gastropoda: Turbinidae). Journal of the World Aquaculture Society 40.

**Scheltema, R. S.** 1967. The relationship of temperature to the larval development of Nassarius obsoletus (Gastropoda). Biological Bulletin 132:253–265.

**Scheltema, R. S.** 1971. Larval dispersal as a means of genetic exchange between geographically separated populations of shallow-water benthic marine gastropods. Biological Bulletin 140:284–322.

**Struhsaker, J. W., and J. D. Costlow.** 1968. Larval development of Littorina picta (Prosobranchia, Mesogastropoda), reared in the laboratory. Journal of Molluscan Studies 38:153–160.

**Switzer-Dunlap, M., and M. G. Hadfield.** 1977. Observations on development, larval growth and metamorphosis of four species of Aplysiidae (Gastropoda: Opisthobranchia) in laboratory culture. Journal of Experimental Marine Biology and Ecology 29:245–261.

**Zhang, L., J. Xia, P. Peng, H. Li, P. Luo, and C. Hu.** 2013. Characterization of embryogenesis and early larval development in the Pacific triton, Charonia tritonis (Gastropoda: Caenogastropoda). Invertebrate Reproduction & Development 57:237–246. https://doi.org/10.1080/07924259.2012.753472
